# MicroRNA-153 impairs presynaptic plasticity by blocking vesicle release following chronic brain hypoperfusion

**DOI:** 10.1186/s12964-020-00551-8

**Published:** 2020-04-06

**Authors:** Mei-Ling Yan, Shuai Zhang, Hong-Mei Zhao, Sheng-Nan Xia, Zhuo Jin, Yi Xu, Lin Yang, Yang Qu, Si-Yu Huang, Ming-Jing Duan, Meng Mao, Xiao-Bin An, Chandan Mishra, Xin-Yu Zhang, Li-Hua Sun, Jing Ai

**Affiliations:** Department of Pharmacology (The State-Province Key Laboratories of Biomedicine-Pharmaceutics of China), College of Pharmacy of Harbin Medical University, Harbin, 150086 Heilongjiang Province China

**Keywords:** Chronic brain hypoperfusion, Presynaptic plasticity, *microRNA-153*, Synaptic vesicle fusion, Low cerebral blood flow

## Abstract

**Background:**

Chronic brain hypoperfusion (CBH) is closely related to Alzheimer’s disease (AD) and vascular dementia (VaD). Meanwhile, synaptic pathology plays a prominent role in the initial stage of AD and VaD. However, whether and how CBH impairs presynaptic plasticity is currently unclear.

**Methods:**

In the present study, we performed a battery of techniques, including primary neuronal culture, patch clamp, stereotaxic injection of the lentiviral vectors, morris water maze (MWM), dual luciferase reporter assay, FM1–43 fluorescence dye evaluation, qRT-PCR and western blot, to investigate the regulatory effect of *miR-153* on hippocampal synaptic vesicle release both in vivo and in vitro. The CBH rat model was generated by bilateral common carotid artery ligation (2VO).

**Results:**

Compared to sham rats, 2VO rats presented decreased field excitatory postsynaptic potential (fEPSP) amplitude and increased paired-pulse ratios (PPRs) in the CA3-CA1 pathway, as well as significantly decreased expression of multiple vesicle fusion-related proteins, including SNAP-25, VAMP-2, syntaxin-1A and synaptotagmin-1, in the hippocampi. The levels of *microRNA-153* (*miR-153*) were upregulated in the hippocampi of rats following 2VO surgery, and in the plasma of dementia patients. The expression of the vesicle fusion-related proteins affected by 2VO was inhibited by *miR-153*, elevated by *miR-153* inhibition, and unchanged by binding-site mutation or miR masks. FM1–43 fluorescence images showed that *miR-153* blunted vesicle exocytosis, but this effect was prevented by either 2′-*O*-methyl antisense oligoribonucleotides to *miR-153* (AMO-153) and miR-masking of the *miR-153* binding site in the 3′ untranslated region (3’UTR) of the *Snap25, Vamp2, Stx1a* and *Syt1* genes. Overexpression of *miR-153* by lentiviral vector-mediated *miR-153* mimics (lenti-pre-*miR-153*) decreased the fEPSP amplitude and elevated the PPR in the rat hippocampus, whereas overexpression of the antisense molecule (lenti-AMO-*153*) reversed these changes triggered by 2VO. Furthermore, lenti-AMO-*153* attenuated the cognitive decline of 2VO rats.

**Conclusions:**

Overexpression of *miR-153* controls CBH-induced presynaptic vesicle release impairment by posttranscriptionally regulating the expression of four vesicle release-related proteins by targeting the 3’UTRs of the *Stx1a*, *Snap25*, *Vamp2* and *Syt1* genes. These findings identify a novel mechanism of presynaptic plasticity impairment during CBH, which may be a new drug target for prevention or treatment of AD and VaD.

**Video Abstract**

**Graphical abstract:**

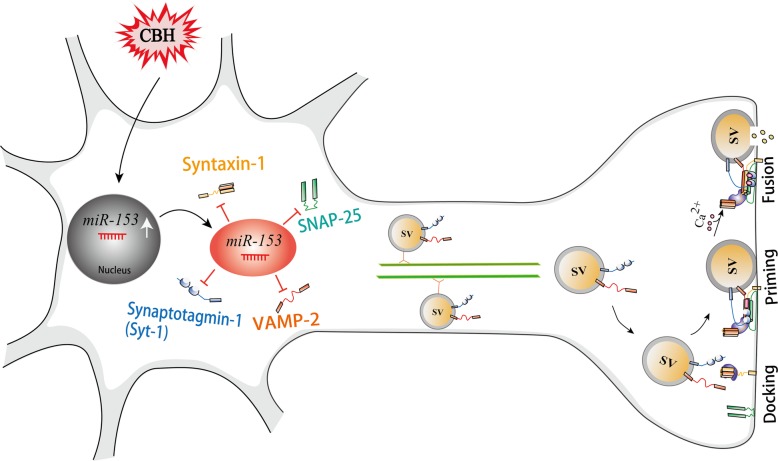

## Background

Recently, chronic brain hypoperfusion (CBH) has been considered serving as a key predictor of the conversion of mild cognitive impairment (MCI) to Alzheimer’s disease (AD) and vascular dementia (VaD) [1]. Previous studies have documented that CBH can produce cognitive decline by affecting many pathological processes including white matter attenuation, neuronal death, neuroinflammation, oxidative stress and even the formation of Aβ and the hyperphosphorylation of Tau [1–6]. Synaptic pathology plays a prominent role in the initial stage of AD and VaD [7–9]. CBH also attenuates synaptic plasticity by changing the expression of several proteins and remodeling synaptic dendrites and spines [10, 11], however, whether and how CBH impairs presynaptic plasticity is currently unclear.

Presynaptic plasticity in chemical synapses is based on neurotransmitter release through synaptic vesicle exocytosis, which relies on highly conserved protein machinery. Neurotransmitter release is well understood to be dependent on the fusion of presynaptic vesicles with presynaptic membranes. This process is controlled by the interaction between the Ca^2+^ sensor protein synaptotagmin-1 (Syt1) and the soluble *N*-ethylmaleimide-sensitive factor attachment receptor proteins complex (SNAREs), which comprises vesicle associated membrane protein 2 (VAMP-2), synaptosomal-associated protein 25 (SNAP-25) and syntaxin-1 [12, 13]. Other important proteins that are required for exocytosis, including Munc-18, Munc-13 and complexins, have been found to collaborate with SNAREs to enhance the fusion probability between vesicles and presynaptic membranes [14, 15]. Importantly, the expression of SNAP-25, VAMP-2, syntaxin-1 and Syt1 has been reported to be lower in postmortem tissues from AD and VaD patients than in those from healthy controls [16–19]. Whether CBH directly affects presynaptic plasticity by interrupting this highly conserved and elaborate protein machinery is unclear.

Previous studies have reported that microRNAs (miRNAs) could regulate presynaptic plasticity by targeting various vesicle-related proteins. For example, *miR-137* impaired vesicle release in a mouse model of schizophrenia [20]; *miR-34c* was reported mediate amyloid-beta (Aβ)-induced synaptic failure by targeting VAMP2 [21]; knockdown of *miR-135a* triggered an early stress response by disturbing presynaptic vesicle release [22]; *miR-153* was found to be a contextual fear-induced miRNA in the dentate gyrus [23] and was reported to regulate vesicle release in zebrafish [24]. However, these studies did not analyze CBH-induced changes in miRNA levels or their function in presynaptic plasticity.

Here we show that CBH obstructs presynaptic vesicle fusion with presynaptic membrane via *miR-153*-mediated downregulation of multiple synaptic vesicle-related proteins. Therefore, we consider that *miR-153* may be a new drug target for the treatment of dementia.

## Methods

### Animals

Male Sprague-Dawley (SD) rats (5 ~ 6 months old) were obtained from the Animal Center of the Second Affiliated Hospital of Harbin Medical University (Harbin, Heilongjiang Province, China). All animals for experiments were housed at 23 ± 1 °C with 55 ± 5% humidity and maintained on a 12-h dark-light artificial cycle (lights on at 7:00 AM) with food and water available ad libitum. Samples for quantitative real-time PCR (qRT-PCR) and western blot assay were obtained from the hippocampi and/or cortices of rats after they had been anaesthetized with 10% chloral hydrate (500 mg/kg, intraperitoneal, Aladdin, Cat. No. C104202, Shanghai, China) followed by confirmation of death by exsanguination. Tissues for primary neuron culture were obtained from neonatal SD rats after administration of 20% isoflurane and confirmation of death by cervical dislocation. All animal procedures were approved by the Institutional Animal Care and Use Committee at Harbin Medical University (No. HMUIRB-2008-06) and the Institute of Laboratory Animal Science of China (A5655–01). All procedures were conformed to the Directive 2010/63/EU of the European Parliament.

### Construction of lentivirus vectors

Lenti-*pre-miR-153*, the *miR-153-3p* overexpression plasmid (RmiR6060-MR03, GeneCopoeia, Guangzhou, China) or negative control (NC) using scrambled RNA oligonucleotides of *miR-153* (lenti-mis-pre-*miR-153-3p*) (CmiR0001-MR03, GeneCopoeia, Guangzhou, China) were generated using the pEZX-MR03 lentiviral transfer vector; while, the *miR-153* inhibitor plasmid lenti-AMO-*153-3p* or NC of AMO-153, using scrambled RNA oligonucleotides (leni-mis-AMO-*153-3p*), was generated using pEZX-AM03 (RmiR-AN0214-AM03, GeneCopoeia, Guangzhou, China). The titers of these RNA oligonucleotide vectors used for experiments were 1 × 10^8^ transducing U/mL.

### Permanent, bilateral common carotid artery occlusion (2VO) in the rat

The method used for preparation of 2VO rats has been describedin previous studies [3, 25] (www.bio-protocol.org/e2668). Briefly, after being anaesthetized by 10% chloral hydrate (300 mg/kg) and maintained under anaesthesia using 0.5–1.0% isoflurane, rats were placed on an electric heating pad (Dongxiyi, Cat. No. wi95919, Beijing, China) to maintain body temperature at 37 °C. Then the fur around the neck was removed using an electric shaver and the area was sterilized by a 75% alcohol cotton ball. A 2-cm incision was made above the manubrium along the anterior midline of the neck. The bilateral common carotid arteries of rats were exposed by vertical separation of the omohyoideus muscle and then carefully separated from the vagus and aortic depressor nerve followed by permanent ligation with 3–0 silk suture (Jinhuan, model: 3–0, China). After the surgical procedures, all the anterior cervical muscles were returned to their original location. To avoid potential postoperative infection, the wounds were washed before closing with 20 mg/mL gentamycin sulfate solution (Sangon Biotech, Cat. No. B540724). The wounds were then sutured and rats were allowed to recover from anaesthesia before being returned back to their cages.

### Stereotaxic injection of the lentiviral vectors

After anaesthesia, rats were placed on a stereotaxic frame (RWB Life Science Co. Ltd., China), and the skull was exposed. Then, 2 μL (10^8^ TU/mL) lenti-*pre-miR-153-3p*, lenti-2′-*O*-methyl antisense oligoribonucleotides to *miR-153* (AMO-153), lenti-mis-pre-*miR-153-3p* and/or leni-mis-AMO-*153-3p* was injected into the bilateral CA1 region of the hippocampus using a 5-μL Hamilton syringe with a 33-gauge tip needle (Hamilton, Bonaduz, Switzerland) at a rate of 0.5 μL/min. The injection coordinate relative to the bregma was as follows: anteroposterior (AP), − 3.60 mm; mediolateral (ML), ±2.30 mm; dorsoventral (DV), − 3.00 mm below the surface of the dura using coordinates derived from the atlas of Paxinos and Watson. The needle was maintained in the place for another 2 min after injection and then withdrawn very slowly to prevent backflow of the solution. Finally, the skin incision was sutured, and the animal was returned to its housing after recovering from anaesthesia. Subsequent experiments were performed 8 weeks after virus injection [3].

### Morris water maze (MWM)

The maze consisted of a black circular pool of 2.0 m diameter, filled with opaque water (25 ± 1 °C) via the addition of black food pigment. A submerged escape platform (20 cm in diameter, top surface 2.0 cm below water level) was located in the centre of the first quadrant. Before training, the pupillary light reflex of all rats was tested, and those with impaired pupillary light reflex were excluded from the experiment to avoid the influence of the animal’s vision on the test. For cued training (three trials per day for 5 d), the rats were released into the water facing the sidewalls, and each rat was allowed 120 s to find the platform, if the rats did not find it in the time allowed, they were guided to the platform and permitted to rest for at least 20 s. After the last cued trial of day 5, the platform was removed from the pool, and each rat was tested on one 120-s swim probe trial on day 6. Escape latency (s) and the number of platform crossings were monitored using an online DigBehav-Morris Water Maze Video Analysis System (Mobile Datum Software Technology Co. Ltd., Shanghai, China) [3, 26].

### Functional magnetic resonance imaging (fMRI) measurements

After rats were anaesthetized with chloral hydrate (300 mg/kg,i.p.), a dose of 0.06 mmoL/kg of Dimeglumine gadopentetate was injected into the rat’s tail vein, and an animal brain coil was used for T2-weighted imaging to acquire fMRI measurements using a 3.0-T animal MIR scanner (PHILIPS: ACHIEVA 3.0-TX) with fast spin-echo plus sequences. The parameters were follows: data matrix = 100 × 92, repetition time (TR) = 2534 ms, effective echo time (TE) = 40 ms, echo train length = 27, field of view = 0.8 × 0.25 cm, twenty-five 1-mm slices, and four signal averages [27].

### 2,3,5-Triphenyltetrazolium chloride (TTC) staining

After the rats were anaesthetized, the brains of rats were immediately removed and cut into 2-mm sections, which were then immersed sequentially into a phosphate-buffered 2% TTC solution at 37 °C for 30 min, and then fixed in phosphate-buffered 4% paraformaldehyde (PFA) solution at 4 °C [3].

### Electrophysiology

#### Hippocampal slice preparation

The rats were anaesthetized with 20% urethane intraperitoneally and decapitated. After the skull was exposed, the whole head was removed and carefully placed on ice-cold dissection buffer N-methyl-D-glucamine (NMDG) solution in mM: 93 NMDG, 2.5 KCl, 10 MgSO_4_, 1.2 NaH_2_PO_4_, 30 NaHCO_3_, 20 HEPES, 0.5 CaCl_2_, 25 D-glucose, 5 sodium-ascorbate, 3 sodium-pyruvate, 2 thiourea and 2 N-acetyl-L-cysteine, with pH 7.3–7.4 adjusted by NaOH or HCl and saturated with O_2_ (95%)/CO_2_ (5%) carbogen mixture for 10 s. After the transverse hippocampal slices (400 μm thick) were prepared using a Leica VT1200S microtome (Leica, Nussloch, Germany), the slices were transferred into NMDG solution at 28 °C for a 3-min recovery, and sequentially maintained in an O_2_ (95%)/CO_2_ (5%)-saturated incubation solution containing (in mM): 124 NaCl, 2.5 KCl,1.2 NaH_2_PO_4_, 24 NaHCO_3_, 2 MgSO_4_, 2 CaCl_2_, 5 HEPES, 2 N-acetyl-L-cysteine, and 12.5 D-glucose (pH 7.3–7.4) for at least 1 h at 33 ± 2 °C. Then the slices were maintained in standard artificial cerebrospinal fluid (ACSF) solution at a constant flow (2 ~ 3 mL/min) and constant temperature of 35 ± 2 °C with the help of a temperature controller (TC-324C, Warner Instruments). The ACSF contained the following (in mM): 124 NaCl, 2.5 KCl, 1.2 NaH_2_PO_4_, 24 NaHCO_3_, 2 CaCl_2_, 2 MgSO_4_, 5 HEPES and 10 D-glucose [28].

#### Extracellular recordings

Concentric bipolar microelectrodes (CBARC75, FHC, USA) were placed in the Schaffer collateral (SC) domain of CA3 300 μm away from recording pipettes, which were placed in the stratum radiatum of CA1. Recording pipettes were pulled from borosilicate glass (BF100–58-10, Sutter Instrument) with resistances of 2–3 MΩ when filled with a NaCl (3 mol/L) solution. The field excitatory postsynaptic potential (fEPSP) of CA3-CA1 was evoked by current stimulation of 0.033 Hz with stimulation steps of 20- to 160- μA (10- steps, 100 ms) using a stimulatory isolator (ISO-Flex (AMPI, Jerusalem, Israel), controlled by a Master-8 pulse generator (AMPI, Jerusalem, Israel). The stimulus intensities, which corresponded to that necessary to evoke ~ 50% of the maximum amplitude of fEPSP, were used for the next synaptic plasticity evaluation [29]. Analogue signals were bypass filtered and digitized at 6 kHz using Digidata 1550A and pClamp10 software (Molecular Devices, US). Paired-pulse facilitation (PPF) in hippocampal CA1 was recorded in separate slices and measured by evoking fEPSP with varying interpulse intervals from 20 to 500 ms.

*Signal analysis* Off-line analysis was performed using Clampfit software (Molecular Devices, US). The basic synaptic transmission was assessed by the normalized amplitude of fEPSP calculated by the amplitude of each fEPSP relative to the amplitude evoked by 20 μA stimulation. Presynaptic vesicle release was evaluated by PPF which is expressed paired-pulse ratio (PPR): fEPSP2 amplitude/fEPSP1 amplitude [30].

### Extraction of synaptosomes

After anaesthetization and perfusion with chilled normal saline, rats were sacrificed with 10% chloral hydrate (500 mg/kg), and the hippocampi were quickly removed and immediately placed on an ice-cold plate, followed by suspension in 10% (w/v) 320 mM sucrose HEPES buffer and homogenization with a grinding rod. The HEPES buffer contained the following (in mM): 145 NaCl, 5 KCl, 2 CaCl_2_, 1 MgCl_2_, 5 glucose and 5 HEPEs buffer with pH 7.3–7.4. Furthermore, the homogenate was centrifuged at 4–8 °C for 10 min at 600×g. The supernatant was then diluted at a 1:1 ratio with 1300 mM sucrose HEPES buffer to yield a suspension at a final concentration of 800 mM sucrose, and centrifuged at 4–8 °C for 15 min at 12000×g. The supernatant was discarded. The pellet consisting of synaptosomes was washed twice with HEPES buffer and centrifuged at 12,000×*g* for 15 min at 4–8 °C to wash out the impurities [31]. To obtain the synapse proteins for western blot experiments, the resulting synaptosomal preparation was disassociated in RIPA buffer with 0.2% TritonX-100 and 1% protease inhibitor. After standing for 30 min on ice, the suspension was further centrifuged at 20,000×*g* for 30 min at 4–8 °C.

### FM1–43 fluorescent dye evaluation

After the neonatal rat hippocampal and cortical neurons (NRNs) were cultured for 10–14 d in cover glasses, they were incubated with FM 1–43 fluorescent dye (T35356, Invitrogen, Oregon, USA) for 3 min at room temperature to allow FM1–43 to bind to the outer membrane of NRNs. Next, 70 mM KCl was added to the FM1–43 loaded NRNs for 3 min at room temperature to allow for the internalization of FM1–43 dye by endocytosis. After this, the NRNs were washed gently with 37 °C 0.9% NaCl to remove the extracellular FM1–43 dye. Finally, 70 mM KCl was added again to elicit synaptic vesicle exocytosis. The FM1–43 fluorescent dye signal was monitored by confocal microscopy (Olympus FV1000, Japan), and fluorescence images were acquired every 1 s [32, 33].

### Primary hippocampal and cortical neuron culture

Rat pups from postnatal days 1–3 (P_1–3_) were first anaesthetized with isoflurane and decapitated, and then the hippocampi and cortices were removed and placed in cold phosphate-buffer solution (PBS, Solarbio, Cat. No. P1010, Beijing). After dissection and trituration, tissues were digested in 0.25% trypsin digestive solution (Beyotime, Cat. No. C0201, Shanghai, China) for 15 min. Single cells were removed after terminating digestion using culture media containing DMEM (HyClone, Cat. No. SH30022.01, Logan, UT, USA) with 10% fetal bovine serum (FBS, HyClone, SH30070.02, Logan, UT, USA) and 1% penicillin-streptomycin (Beyotime,Cat. No. C0222, Shanghai, China). The cells were then plated on cell plates precoated with 10 μg/mL poly-D-lysine (PDL, No. P0899 Sigma, Saint Louis, CA, USA) and cultured in culture media containing neurobasal medium (Invitrogen, Cat. No. 21103049, Carlsbad,USA) with 2% B27 supplement (Invitrogen, Cat. No. 17504004, Carlsbad, CA, USA) without FBS. After 3 d, the neurons were treated with 5 μM cytosine arabinoside (Sigma, Cat. No. C8768, USA) to inhibit astrocyte proliferation. For the western blot and qRT-PCR experiments, the neurons were used 5–7 d after plating. For the FM1–43 dye experiments, the neurons were plated on cover glass precoated with 10 μg/mL PDL and cultured for 12 d. Half the volume of culture media was changed every three days [3].

### Dual luciferase reporter assay

Plasmid construction was performed according to the method previously described [3, 34]. Genome DNA templates were first extracted from rat tails with Wizard® genome DNA purification kit (Promega, Cat. #A1125), and DNA electrophoresis was used to verify the quality of extracted genomic DNA using DL2000DNA marker (Fig. S1a). The sequences of the full-length 3′ untranslated region (3’UTR) and mutant 3’UTR of the *Snap25, Vamp2, Stx1a* and*, Syt1* genes *3* are shown in Additional file 1. Amplification of the acquired genomic DNA was performed by PCR and the PCR products were recollected with a DNA gel extraction kit (Dongsheng Biotech) (Fig. S1a-e). The primers for the amplification of the full-length 3’UTR of the *Snap25, Vamp2, Stx1a,* and *Syt1* genes are shown in Table S1. Next, the purified PCR products were incubated with psiCHECK-2 vectors using the *XhoI* and *NotI* enzyme for enzymatic digestion. The enzyme digestive products were purified on an agarose gel with a DNA Gel extraction kit and ligated into psiCHECK-2 vectors using T4 DNA ligase (TaKaRa, D2011A). The ligated products were transformed into *E.coli* DH5α competent cells. The positive recombinant clones were screened by agarose gel electrophoresis and BLAST analysis of cloned DNA sequences relative to the 3’UTRs of the genes published in NCBI was employed to verify vector construction (Fig. S2).

Mutagenesis of nucleotides was carried out using direct oligomer synthesis for the 3’UTR region of *Syt1, Stx1a, Snap25* and *Vamp2*. Point mutations were introduced into a possible *miR-153* binding site located in the 3’UTR region. Plasmid construction for carrying the mutated 3’UTRs of these four genes were constructed using the same method as that used for wild-type plasmid construction. The PCR primers for the mutagenesis of the 3’UTRs of these four genes are shown in Table S1.

HEK293T cells (2 × 10^4^ cells) were inoculated on 24-well plates at 50% ~ 60% confluences. These cells were then transfected with 20 μmol/L *miR-153* mimics, AMO- *153*, or mis- *miR-153* siRNAs as well as 0.5 μg psi-CHECK™-2-target DNA (Renilla luciferase vector) and 1 μL blank plasmid using Lipofectamine 2000 (Invitrogen, USA) transfection reagent according to the manufacturer’s instructions. After 48 h of transfection, luciferase activities were measured with a dual luciferase reporter assay kit (Cat. No. E1910, Promega, USA) and luminometer (GloMax™ 20/20, Promega, USA); Nucleotide-substitution mutagenesis was carried out using direct oligomer synthesis for the 3′ UTRs of *Stx1a*, *Snap25*, *Vamp2* and *Syt-1*. All constructs were sequence verified.

### Transfection procedures

After six days of cell culture, 75 pmoL/mL *miR-153* mimics and/or AMO-153, oligodeoxynucleotides (ODNs), scrambled *miR-153* or scrambled AMO-153 (Table S2) were transfected into cultured neonatal hippocampal and cortical neurons with X-treme GENE siRNA transfection reagent (catalog#04476093001, Roche, USA) according to the manufacturer’s instructions. Forty-eight hours after transfection, cells were collected for subsequent total RNA isolation, protein purification or FM1–43 staining. Transfection was performed at 12 d for the FM1–43 dye experiments [3].

### Immunofluorescence detection

After 48 h, the cultured cells were fixed in 4% paraformaldehyde for 30 min. After the cells were blocked, they were incubated with the Tuj1 antibody (Cat.#T8578; 1:200; Sigma, USA) to label the neurons and then the cultured cells on the glass were washed and incubated with the secondary antibodies conjugated to Alexa Fluor 488 (Invitrogen) for 1 h at 37 °C. After incubation with secondary antibody and DAPI as usual, neurons were mounted on coverslips to obtain confocal images by FluoView™ FV300 (Olympus) using × 60 objective with the same condition at a resolution of 1024 × 1024 pixels (12 bit) [27].

### qRT-PCR

Total RNA was purified with the Trizol reagent (Invitrogen, Carlsbad, CA, USA) according to the manufacturer’s instructions. The extracted RNA was then reverse transcribed into cDNA according to the introduction of the ReverTra Ace® qPCR RT Kit (Code: FSQ-101, Osaka, Japan). The *miR-153* level was assessed using the FastStart Universal SYBR® Green Master kit (Roche Diagnostics GmbH, Mannheim, Germany). U6 was used as an internal control of miRNAs and actin was selected as the internal reference mRNA. The qRT–PCR probes and primers for *miR-153*, *miR-137*, *miR-34c*, *miR-135a*, and *U6* were designed by Invitrogen (Table S3). QRT-PCR was performed on a LightCycle® 96 Instrument (Roche Diagnostics GmbH, Mannheim, Germany), and the protocol was as follows: 1.preincubation: 95 °C, 10 min; 2.amplification: (1) denaturation: 95 °C, 15 s;(2) anneal: 60 °C, 30 s; 3) extension: 72 °C, 30 s;3. cooling: 37 °C, 30 s. Repeat “2” for 40 cycles. The results were normalized against the internal control using the δ–δ CT method [3].

### Western blot

The concentrations of the protein extracts were measured using the Bio-Rad Protein Assay kit (Bio-Rad, Hercules, CA, USA) with bovine serum albumin standards. Protein samples fractionated on a 10% sodium dodecyl sulfate polyacrylamide gel electrophoresis gel were transferred to a nitrocellulose membrane. Table S4 displays all of the primary antibodies used in this study. Western blot bands were captured using the Odyssey Infrared Imaging System (LI-COR Biosciences, Lincoln, NE, USA) and quantified using Odyssey version 3.0 software by measuring the band intensity (area – optical density (OD)). Protein expression in each group was normalized to the internal control β-actin (1:1000, G8795, Sigma).

### Human blood sample preparation

Vascular dementia patients (between 70 ~ 90 years old), from the Second Affiliated Hospital of Harbin Medical University, were recruited for this experiment based on the criteria of the National Institute of Neurological and Communicative Disorders (NINCDS) (Sarazin, etal., 2012). Written consent was obtained from all subjects, and the study protocol was approved by the Ethics Committee of Harbin Medical University (HMUIRB20140029). To prepare plasma samples for miRNA evaluation, whole blood samples (2.5 mL per patient) were collected from subjects via a direct venous puncture into tubes containing sodium citrate and centrifuged at 1000×g for 5 min, and then the supernatant (plasma) was carefully transferred into an RNase-free tube for RNA extraction [35].

### Statistical analysis

All data are represented by the mean and standard error of the mean (s.e.m). Each data set was analysed for its ability to meet the statistical assumptions for equality of the variance. The independent sample test was calculated using the Levene variance equality test. If *P* > 0.05, independent Student’s t-test was used for the comparison between two groups; if *P* < 0.05, the Kruskal-Wallis rank sum test was performed. One-way ANOVA was performed for the comparison among multivariate groups, and post hoc analyses of significant main effects were further examined using Fisher’s procedures for learning systems design (PLSD) tests. For repeated measures data, Mauchly’s test of sphericity was first performed to evaluate the relationship among the repeatedly measured data. If *P* > 0.05, a general linear model was selected for further analysis; if *P* < 0.05, Greenhouse-Geisser corrected results or multivariate ANOVA was used for further analysis of multiple comparisons. All statistical analyses were performed in SPSS software (version 11).

## Results

### CBH impairs presynaptic function in rats

**Fig. 1:**
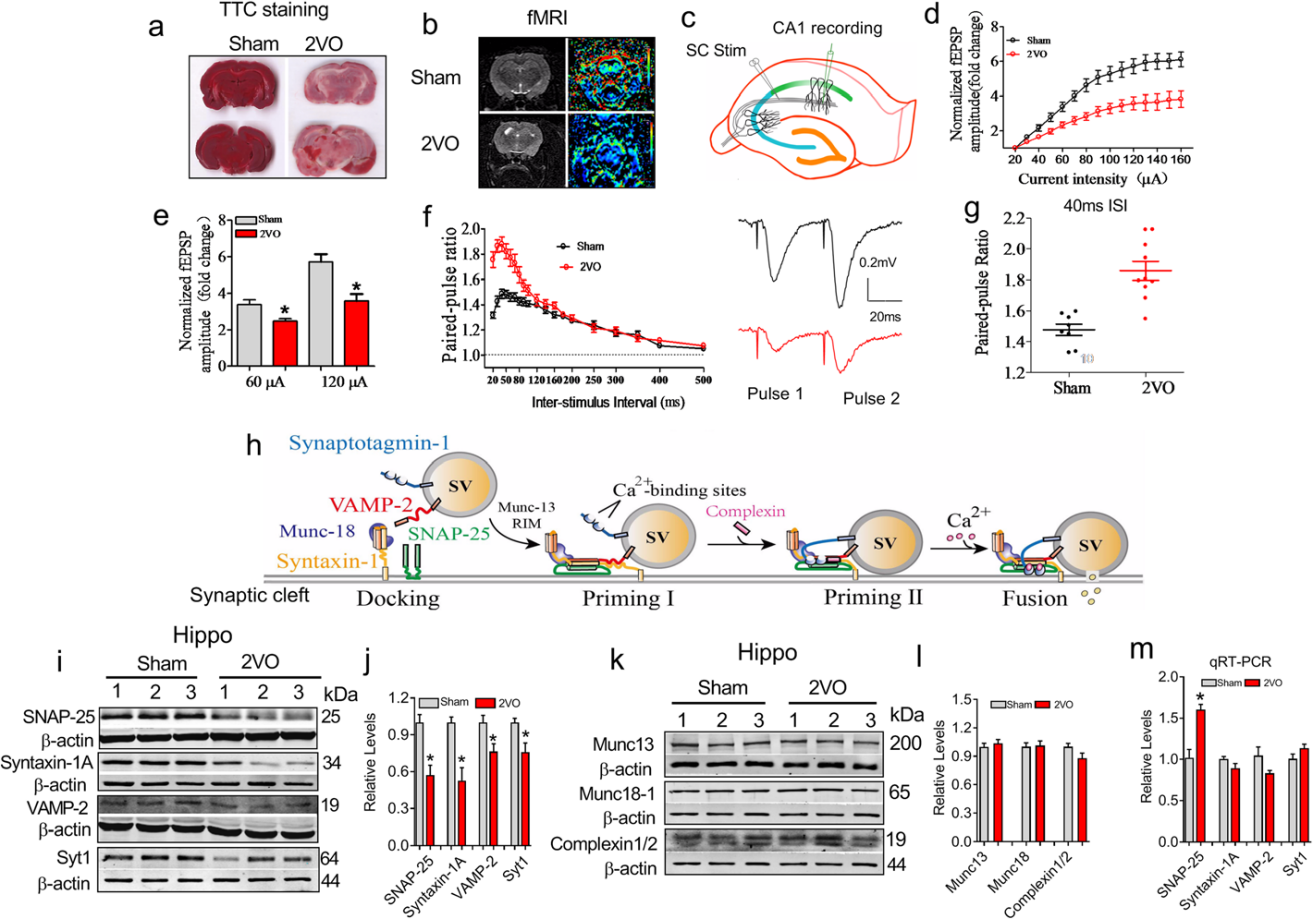
Impairment of presynaptic vesicle release in CBH rats 8 W following 2VO surgery. **a** TTC staining was used to identify brain ischaemia in rats at 8 W after 2VO. Red represents normal tissue and white represents infarct tissue. **b** fMRI brain images of rats at 8 W after 2VO. fMRI, functional magnetic resonance imaging. **c** Schematic diagram showing the placement of the recording (CA1) and stimulating electrodes (CA3) in hippocampal slices. **d** Comparison of I-O curves between sham and 2VO rats. F _(14, 196)_ = 15.011, *P* < 0.002. *n* = 8 rats. **e** Fold-change differences in the fEPSP amplitude between the 2VO and sham groups. P_Levene_ = 0.713, Student’s t-test: *P* = 0.003. *n* = 8. **f** Scatter plot summarizing the PPR in sham and 2VO rats. Right Insertion: sample of fEPSP traces in the sham and 2VO rats with a 40 ms ISI stimulated by 60 μA. *χ*^*2*^_Mauchly_ = 520.339, F _(18, 379)_ = 19.122, *P* < 0.0001. n = 8 slices for sham rats, *n* = 10 for 2VO rats. **g** Scatter dot plot showing the increased PPR in the 2VO rats compared with that in sham rats with a 40 ms ISI. Each dot represents one data point from one slice. P_Levene_ = 0.073, Student’s t-test: *P* < 0.0001. **h** Diagram depicting a molecular model of the synaptic vesicle exocytosis process. **i**-**j** Expression of SNAP-25, syntaxin-1A, VAMP-2 and Syt1 proteins in the hippocampal synaptosomes of 2VO rats. *n* = 6 rats. SNAP-25: *P*_Levene_ = 0.719, Student’s t-test, *P* = 0.0017; syntaxin-1A: *P*_Levene_ = 0.002. Kruskal-Wallis rank sum test: *P* = 0.005; VAMP-2: *P*_Levene_ = 0.843, Student’s t-test, *P* = 0.02; Syt1: *P*_Levene_ = 0.477, Student’s t-test, *P* = 0.015. *P < 0.05. **k**-**l** Protein expression of Munc-13, Munc-18-1 and complexin 1/2 in hippocampal synaptosomes of 2VO rats. Student’s t-test, *P* > 0.05. **m** Relative mRNA levels of SNAP-25, syntaxin-1A, VAMP-2 and Syt-1 in the hippocampi of 2VO rats (Student’s t- test: SNAP-25, *P* = 0.025; syntaxin-1A, *P* = 0.303; VAMP-2, *P* = 0.361; Syt-1, *P* = 0.287). n = 6 rats. *P < 0.05 vs sham

To evaluate presynaptic plasticity in the hippocampi of CBH rats, we performed 2VO surgery on rats [3, 36]. As shown in Fig. 1a and b, similar to our previous study [3], 2VO surgery elicited markedly low cerebral blood flow (CBF) at 8 weeks (8 W), as indicated by both TTC staining and fMRI evaluation. We then prepared brain slices and monitored the fEPSP in the stratum radiatum of CA1 by electrically stimulating the SC of the CA3 pathway (Fig. 1c). Based on the input-output (I-O) curve, compared with the sham group, the 2VO group exhibited reduced fEPSP responses (Fig. 1d, *P* = 0.002). For example, the fEPSP amplitude in the 2VO rat slices was ~ 75% of that observed in the sham controls when the stimulus intensity was 60 μA, and the percentage was further decreased to ~ 60% at 120 μA stimulation (Fig. 1e, *P* = 0.003). To evaluate the presynaptic vesicle release probability, PPF (60 μA) measurements were performed and evaluated by PPR [30]. The PPR in the brain slices of the 2VO group, compared with that of the sham controls, was significantly increased when the interstimulus interval (ISI) ranged from 20 ~ 100 ms (Fig. 1f, *P* < 0.0001). The PPR was 1.48 ± 0.01 in the sham group and 1.85 ± 0.02 in the 2VO rats when the ISI was 40 ms (Fig. 1g, *P* < 0.0001). These findings demonstrate an impaired presynaptic function in the 2VO rats.

Previous elegant studies have demonstrated that synaptic vesicle fusion is a crucial step for presynaptic neurotransmitter release triggered by the influx of Ca^2+^, which is controlled by the core fusion machinery composed of the SNARE-complex and Sec1/Munc18-like (SM) proteins [12, 37] (Fig. 1h). We speculated that synaptic vesicle fusion-related proteins might be changed under CBH conditions. Western blotting experiments showed that the protein levels of all three SNARE-complex proteins (SNAP-25, *P* = 0.0017; syntaxin-1A, *P* = 0.005 and VAMP-2, *P* = 0.02) and the Ca^2+^-sensor protein Syt1 (*P* = 0.015) were significantly lower in the hippocampi of 2VO rats than in those of sham rats (Fig. 1i and j). However, the protein levels of Munc-13, Munc-18-1 and Complexin1/2, coregulators of vesicle exocytosis, were unchanged (Fig. 1k and l, *P* > 0.05). These findings suggest that the decreased protein expression of SNARE complex proteins and Syt1 may participate in the impaired presynaptic vesicle release observed in CBH rats.

### *MiR-153* targets SNARE complex-related genes and the *Syt1* gene

**Fig. 2:**
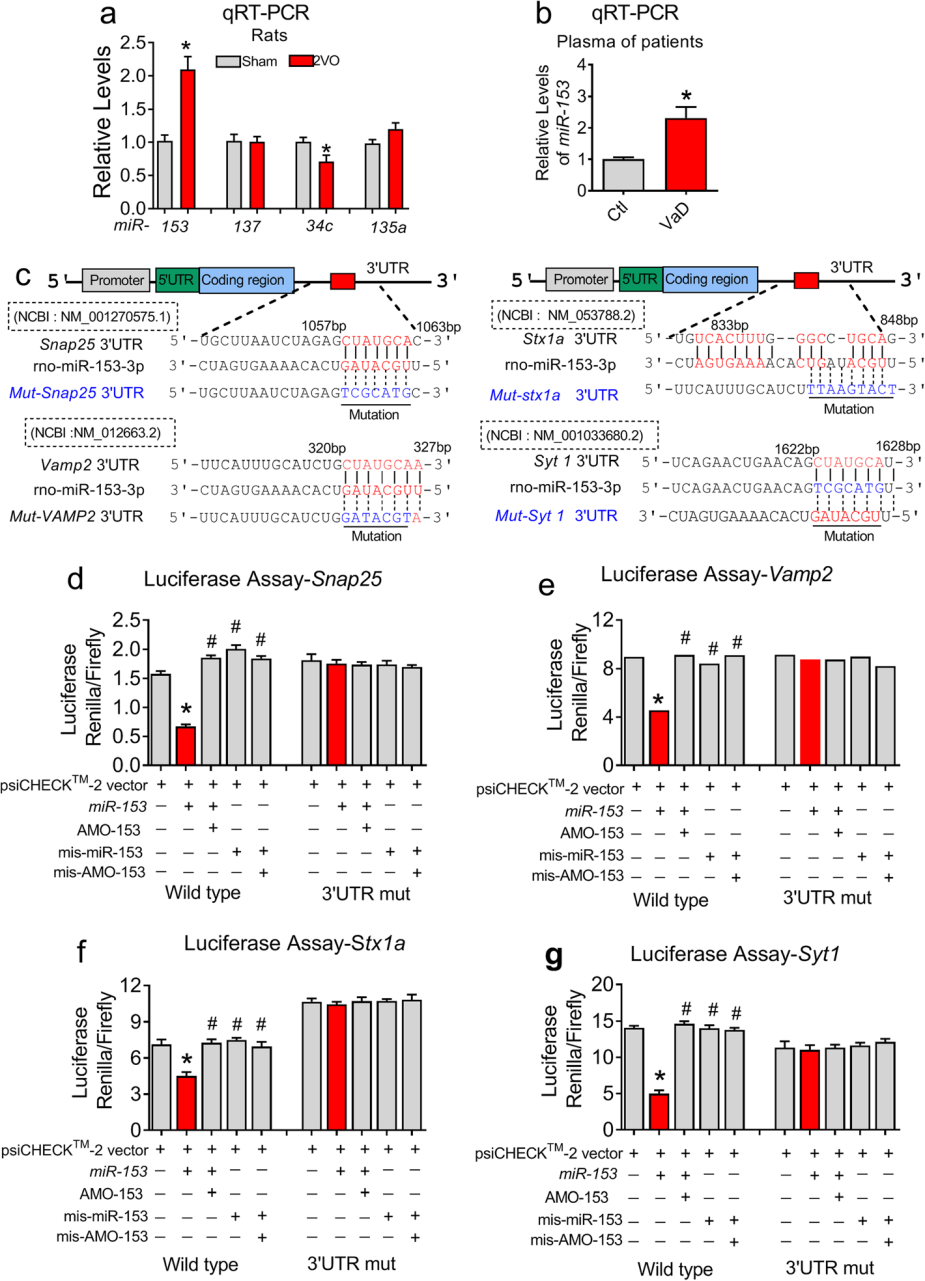
*MiR-153* targets the 3’UTRs of the *Snap25, Vamp2, Stx1a* and *Syt1* genes. **a** The levels of *miR-153, miR-137, miR-34c* and *miR-135a* in the hippocampi of sham and 2VO rats as assessed by qRT-PCR (for *miR-153: P*_Levene_ = 0.008, Student’s t-test: *P* = 0.011), *n* = 6. **b***MiR-153* levels in the plasma of VaD patients as assessed by qRT-PCR (*P*_Levene_ = 0.002, Student’s t-test, *P* = 0.019). VaD: vascular dementia. **c** Complementarity between the *miR-153* seed sequence and the 3’UTRs of *Snap25, Vamp2, Stx1a* and *Syt1* predicted using the TargetScan5.1 algorithm and RNAhybrid database. The gene mutations are underlined. 3’UTR, 3′ untranslated region. **d**-**g** Dual luciferase assay of interactions between *miR-153* and predicted binding sites or mutated binding sites in the 3’UTR of *Snap25* (wild-type: *P*_Levene_ = 0.192, one-way ANOVA: F = 85.093, *P* < 0.0001; 3’UTR mutant (3’UTR mut): *P*_Levene_ = 0.184, one-way ANOVA: F = 0.199, *P* = 0.933), *Vamp2* (wild-type: *P*_Levene_ = 0.168, one-way ANOVA: F = 55.704, *P* < 0.0001; 3’UTR mut: *P*_Levene_ = 0.041, one-way ANOVA: F = 2.561, *P* = 0.104), *Stx1a* (wild-type: *P*_Levene_ = 0.276, one-way ANOVA: F = 119.138, *P* < 0.0001; 3’UTR mut: *P*_Levene_ = 0.0331, one-way ANOVA: F = 0.155, *P* = 0.957) and *Syt1* (Wild-type: *P*_Levene_ = 0.522, one-way ANOVA: F = 825.5700, *P* < 0.0001; 3’UTR mut: *P*_Levene_ = 0.077, one-way ANOVA: F = 1.603, *P* = 0.248) in HEK293T cells. *n* = 3 batches per group. Fisher’s PLSD test was used for the post hoc analyses of the two-group comparisons. *P < 0.05 vs control or psiCHECK™-2 vector; ^#^*P* < 0.05 vs *miR-153*

Compared to the levels in sham rats, the SNARE complex protein and Syt1 protein levels were decreased in 2VO rats, while their mRNA levels were not reduced, indeed, and the level of SNAP25 mRNA was even increased in 2VO rats (Fig. 1m, *P* = 0.025). Therefore, we considered that a posttranscriptional regulatory mechanism might be involved in the observed changes. Multiple miRNAs have been previously reported to regulate synaptic fusion-related proteins at the mRNA level. For example, *miR-153* regulates *Snap25*, *Vamp2*, *Snca*, *Trak2*, *Bsn* and *Pclo* genes at the mRNA level [23]; *miR-137* inhibits complexin-1, Nsf and Syt1 in mouse model of schizophrenia [20]; *miR-34c* targets VAMP-2 [21] and *miR-135a* binds the complexin-1 and complexin-2 genes in the amygdala [22]. To identify which one of the above miRNAs is involved in the CBH induced downregulation of fusion-related proteins in the hippocampi of rats, we performed qRT-PCR test. As displayed in Fig. 2a, compared to the sham rats, 2VO rats exhibited ~ 2-fold increase in the expression of *miR-153* (*P* = 0.011), however, the *miR-137* and *miR-135a* levels were unchanged (*P* > 0.05). In contrast, *miR-34c* expression was reduced in the hippocampi of 2VO rats. These data suggest that the decreased expression of SNAP-25 or VAMP2 in the hippocampi of 2VO rats may result from upregulated expression of *miR-153*, but do not correlate with the observed downregulation of *miR-34c.* Additionally, the decrease in Syt1 protein expression was not associated with *miR-137* expression. Furthermore, to elucidate the potential clinical significance of *miR-153*, 12 male subjects (7 controls and 5 VaD patients) with higher education backgrounds were recruited to our study and assessed by the Mini-Mental State Examination (MMSE), Montreal Cognitive Assessment (MoCA) and Hamilton Depression Scale (HAMD) (Table S5). As shown in Fig. 2b, the levels of *miR-153* in the plasma of VaD patients were significantly higher than those in the plasma from the control group (*P* = 0.019), indicating that *miR-153* is potentially involved in the pathophysiological process of human presynaptic vesicle release disorders. Bioinformatics methods (TargetScan Human 5.1 and RNAhybrid database) were then employed to identify the potential miRNAs regulating Syt1 and syntaxin-1A expression. Surprisingly, we found that *miR-153* alone had conserved binding sites in the 3’UTRs of the *Snap25* and *Vamp2* genes as well as targets in the 3’UTRs of the *Stx1a* and *Syt1* genes (Fig. 2c).

**Fig. 3:**
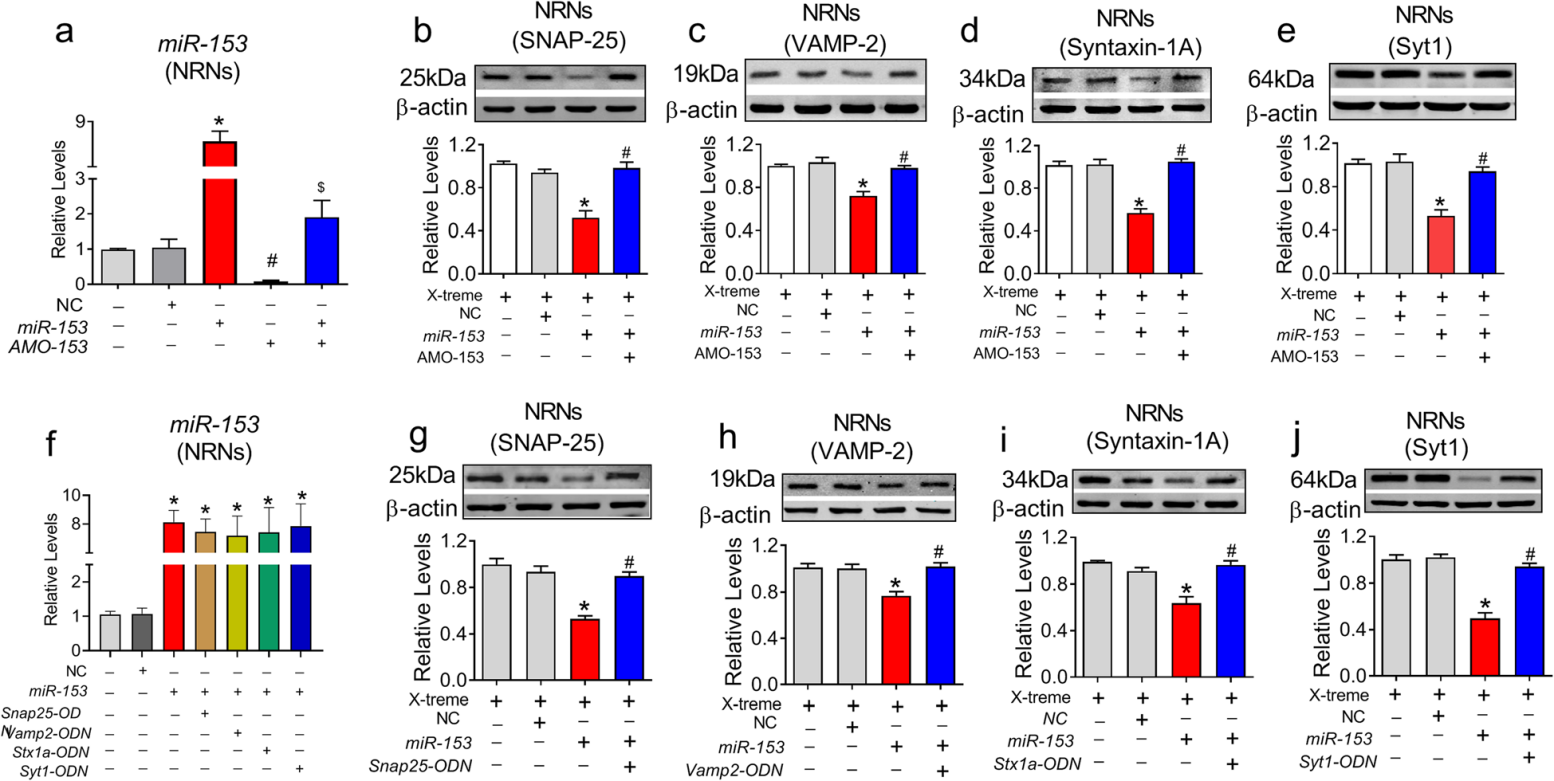
*MiR-153* regulates the expression of vesicle-related proteins. **a***MiR-153* levels after transfection with *miR-153* mimics and AMO-153 assessed by qRT-PCR. *P*_Levene_ = 0.060, one way ANOVA: F = 63.818, *P* < 0.0001; Fisher’s PLSD test was used for post hoc analyses of two-group comparisons. n = 3 batches of neurons per group. **b-e***MiR-153* downregulates the expression of SNAP-25 (*P*_Levene_ = 0.411, one-way ANOVA: F = 12.556, *P* = 0.002), VAMP-2 (*P*_Levene_ = 0.180, one-way ANOVA: F = 9.350, *P* = 0.005), syntaxin-1A (*P*_Levene_ = 0.669, one-way ANOVA: F = 18.496, *P* = 0.001) and Syt1 (*P*_Levene_ = 0.200, one-way ANOVA: F = 10.376, *P* = 0.001) in primary cultured NRNs, and this downregulation was blocked by AMO-153. n = 6 batches of neurons per group. f *MiR-153* levels after ODNs transfection assessed by qRT-PCR. n = 3 batches of neurons per group (*P* < 0.0001). ODN, oligodeoxynucleotides. **g–j** Gene-specific *Snap25-ODN* (*P*_Levene_ = 0.689, one-way ANOVA: F = 13.142, *P* = 0.002), *Vamp2-ODN* (*P*_Levene_ = 0.365, one-way ANOVA: F = 8.235, *P* = 0.008), *Stx1a*-ODN (*P*_Levene_ = 0.124, one-way ANOVA: F = 9.975, *P* = 0.004) and *Syt1-ODN* (*P*_Levene_ = 0.590, one-way ANOVA: F = 23.451, *P* < 0.0001) reversed the *miR-153-*mediated repression of the respective proteins in NRNs. *n* = 4 batches of neurons per group. Fisher’s PLSD test was used for the post hoc analyses of the two-group comparisons. *P < 0.05 vs *NC*; ^#^*P* < 0.05 vs *miR-153*

To better understand how *miR-153* regulates all four genes, we first performed a dual luciferase reporter gene assay to evaluate the binding ability of *miR-153* with these genes. The full-length 3’UTRs of these four genes containing the *miR-153* binding sites were separately cloned, purified, and ligated into the psiCHECK™-2 vector at the site between the *Renilla* luciferase gene and the synthetic poly (A) tail (Fig. S1a-e and S2a-c). Thereafter, the effects of *miR-153* on reporter activities were assessed in HEK293T cells. As predicted, cotransfection of chemically synthesized *miR-153* mimics and the psiCHECK™-2 vector plasmid consistently produced lower Renilla luciferase activities than transfection of the empty psiCHECK™-2 vector plasmid alone (Fig. 2d-g, *P* < 0.0001). The silencing effect of *miR-153* reached ~ 60% on the *Snap25* transcript (Fig. 2d), ~ 50% on the *Vamp2* transcript (Fig. 2e), ~ 35% on the *Stx1a* transcript (Fig. 3f) and ~ 70% on the *Syt1* transcript (Fig. 2g), and these effects could be prevented by cotransfection of the psiCHECK™-2 vector plasmids with AMO-153. However, the transfection of psiCHECK™-2 vector plasmids with scrambled oligoribonucleotides of either mis-*miR-153* or antisense mis-AMO-153 in all four genes failed to affect luciferase activity (Fig. 2d-g). Furthermore, when we cloned the full-length 3’UTR of the *Snap25* (1057–1063 bp), *Vamp2* (320–327 bp), *Stx1a* (841–848 bp) and *Syt1* (1622–1628 bp) with mutant binding sites (Fig. 2c), the repressive effects of *miR-153* on the luciferase activities of these genes were abolished (Fig. 2d-g, *P* > 0.05). These results suggest that *miR-153* directly target the 3’UTRs of the *Snap25*, *Vamp2*, *Stx1a and Syt1* genes.

To confirm that *miR-153* gain-of-function affects the expression of these four proteins in neurons, chemically synthesized *miR-153* mimics and AMO-153 were successfully transfected into cultured NRNs by the X-treme GENE siRNA transfection reagent (Fig. 3a, *P* < 0.0001 and Fig. S3). The *miR-153* mimics effectively inhibited the expression of all four proteins with an inhibitory efficiency of ~ 48% for SNAP-25 (*P* = 0.002), ~ 47% for Syt1 (*P* = 0.001), ~ 43% for syntaxin-1A (*P* = 0.002) and ~ 28% for VAMP-2 (*P* = 0.005), whereas the scrambled NC failed to affect the levels of these proteins (Fig. 3b – e). In contrast, AMO-153 rescued the downregulation of all proteins elicited by the *miR-153* mimics. Subsequently, to confirm that *miR-153* mediates the downregulation effect on these four proteins by directly targeting the binding sites of the four genes, the miRNA-masking antisense ODNs (miR-masking) technique was employed as previously reported [3]. An ODN is an antisense oligodeoxynucleotide fragment designed to fully base pair to a protein-coding mRNA at the sequence motif spanning the binding site for an endogenous miRNA of interest. Since a miR-mask only acts on the target gene with minimal effects on other target genes that may also be targeted by a same miRNA, the anti-miRNA action of a miR-mask is gene-specific. Based on this mechanism, we designed four *miR-153* masks that could base pair the *miR-153* binding sites in the 3’UTRs of the *Snap25, Vamp2, Stx1a* and *Syt1* genes and labelled these masks *Snap25-ODN, Vamp2-ODN*, *Stx1a-ODN* and *Syt1-ODN*, respectively, and evaluated whether these ODNs could shield the action of *miR-153* on the four genes. As predicted, after successful transfection of the four ODNs (Fig. 3f), these ODNs effectively blocked the repressive effects of *miR-153* on the protein expression of SNAP-25 (*P* = 0.002), syntaxin-1A (*P* = 0.004), VAMP-2 (*P* = 0.008) and Syt1 (*P* < 0.0001) (Fig. 3g-j), highlighting the sequence specificity of the actions of *miR-153*.

### *MiR-153* regulates synaptic vesicle release by targeting SNARE complex-related genes and the *Syt1* gene in vitro

**Fig. 4:**
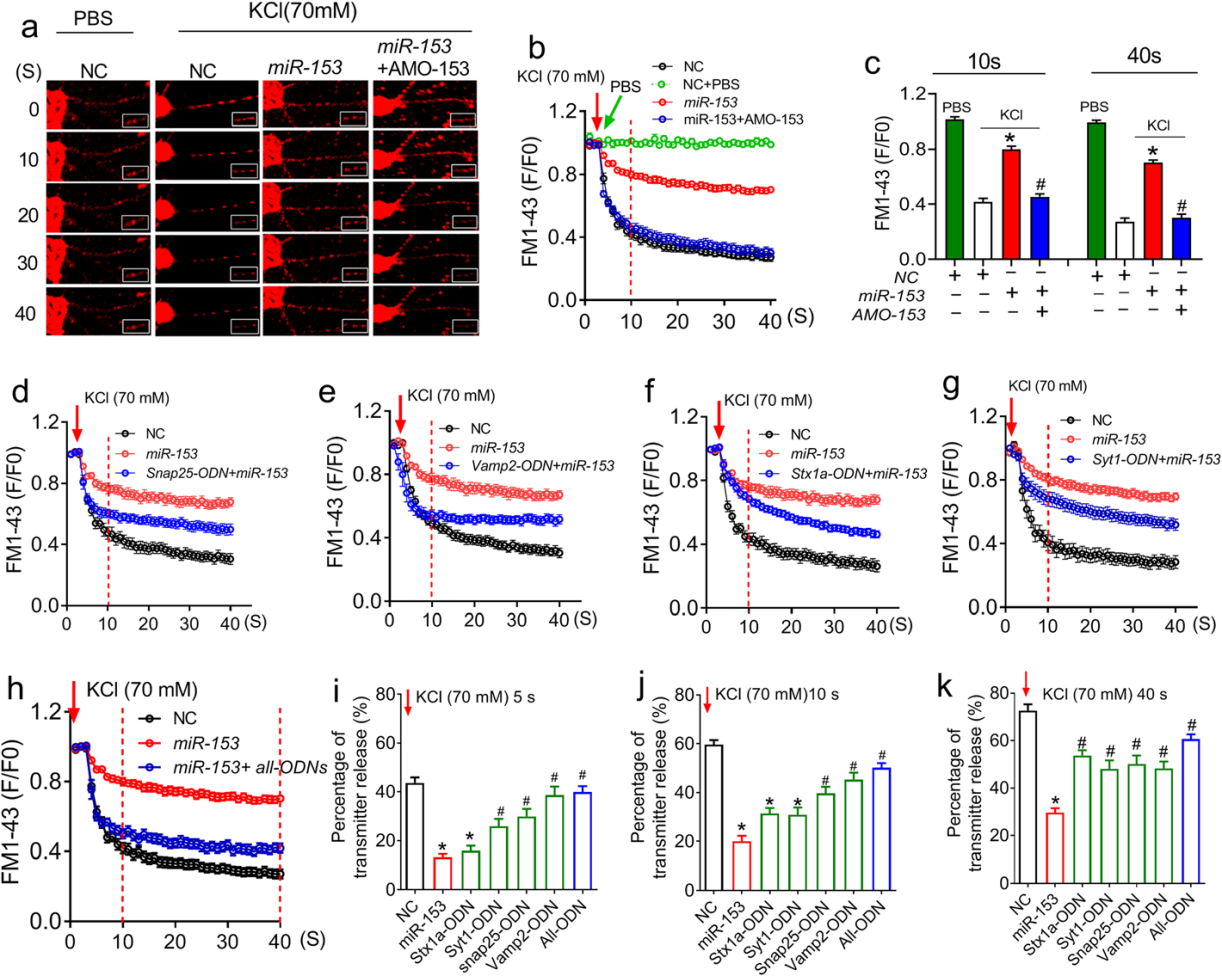
*MiR-153* represses synaptic vesicle exocytosis by targeting vesicle release-related proteins. **a** FM1–43 fluorescent dye images showing presynaptic boutons in neurons before stimulation (t = 0 s) and following PBS or 70 mM KCl stimulation at 10, 20, 30, and 40 s. The insert shows enlarged synaptic boutons from the framed area in the main panels. **b** FM1–43 fluorescent dye imaging analysis (*χ*^*2*^_Mauchly_ = 3068.620, *P* < 0.0001; F_total(39, 2146)_ = 69.973, *P* < 0.0001). **c** Bar graph of FM1–43 staining normalized fluorescence density at 10 s (*P*_Levene_ = 0.330, one-way ANOVA: F = 46.738, *P* < 0.0001) and 40 s (*P*_Levene_ = 0.105, one-way ANOVA: F = 90.788, *P* < 0.0001) following 70 mM KCl stimulation. *n* = 17 synaptic boutons from 3 batches of NRNs in each group. **d**-h FM1–43 fluorescence intensity curve showing the action of *Snap25-ODN* (**d**, F_total (39, 1132)_ = 27.987, *P* < 0.0001), *Vamp2-ODN* (e, F_total (39, 1132)_ = 32.628, *P* < 0.0001), *Stx1a-ODN* (**f**, F_total (39, 1132)_ = 40.414, *P* < 0.0001), *Syt1-ODN* (**g**, F_total (39, 1132)_ = 32.183, *P* < 0.0001) or the four ODNs combined (**h**, F_total (39, 2029)_ = 25.076, *P* < 0.0001) on FM1–43 fluorescent dye exocytosis. **i**-k Statistical analysis of the effects of the SNAP-25, VAMP-2, syntaxin-1A and Syt1 proteins on vesicle release. Bar graph showing the percentage of released vesicles after 70 mM KCl stimulation at 5 s (**i**: *P*_Levene_ = 0.357, one-way ANOVA: F = 15.657, *P* < 0.0001), 10 s (**j**: *P*_Levene_ = 0.027, one-way ANOVA: F = 24.181, *P* < 0.0001) and 40 s (**k**: *P*_Levene_ = 0.142, one-way ANOVA: F = 33.132, *P* < 0.0001). *n* = 12 synaptic boutons from 3 batches of NRNs in each group. Fisher’s PLSD test was used for the post hoc analyses of the two-group comparisons. *P < 0.05 vs *NC*; ^#^*P* < 0.05 vs *miR-153*

To determine whether *miR-153* gain-of-function impairs synaptic vesicle release, we loaded NRNs with FM1–43 fluorescent dye, which can be used to monitor vesicle release by labelling synaptic vesicles [32]. As illustrated in Fig. 4a and b, after treatment with phosphate buffer saline (PBS), the red fluorescent signal of the synaptic boutons was not changed in the NRNs transfected with NC, which was sharply depleted by adding KCl. The F/F0 ratio was reduced to ~ 0.4 at 10 s and further decreased to ~ 0.3 at 40 s. However, the *miR-153* mimics markedly blocked the fast-declining fluorescent signal of the boutons with the F/F0 ratio as high as 0.8 at 10 s and 0.7 at 40 s after KCl stimulation; this effect was fully reversed by the cotransfection with AMO-153 (Fig. 4a-c, *P* < 0.0001). Overall, these data provide evidence that *miR-153* gain-of-function impairs presynaptic vesicle release.

Subsequently, four *miR-153* masks: *Snap25-ODN*, *Vamp2-ODN*, *Stx1a-ODN* and *Syt1-ODN*, were employed to observe the contribution of the four ODNs to the *miR-153-*induced impairment of vesicle release. We found that the effect of *miR-153* on vesicle release was only partially prevented when any single ODN was transfected into NRNs (Fig. 4d-g). Importantly, the effect of *miR-153* on vesicle release within 5 s was virtually entirely dependent on the SNAP-25 and VAMP-2 proteins because *Snap25-*ODN or *Vamp2-*ODN alone almost fully reversed the *miR-153* induced elevation of the F/F0 ratio (Fig. 4d, e and I, *P* < 0.0001). However, within this time window, no effect of *Stx1a-ODN* (Fig. 4f and i) and a weak action of *Syt1*-*ODN* (Fig. 4g and i, *P* < 0.0001) on the F/F0 ratio were observed. Notably, similar to the performance of all four ODNs together, *Vamp2-ODN* alone completely reversed the blunting effect of *miR-153* on vesicle release at 5 s (Fig. 4e, h and i, *P* < 0.0001). Interestingly, as the duration after KCl supplementation increased to 40 s, the preservative effect of *Stx1a-ODN* and *Syt1-ODN* on the *miR-153*-induced impairment of vesicle release was increased and became similar to the effects of *Snap25-*ODN and *Vamp2-*ODN (Fig. 4j and k). These results suggest that the *miR-153* binding sites on the 3’UTRs of these four genes are involved in the impairment of presynaptic vesicle release, and VAMP-2 and SNAP-25 are the rate-limiting proteins.

### *MiR-153* gain-of-function impairs presynaptic vesicle release in vivo

**Fig. 5:**
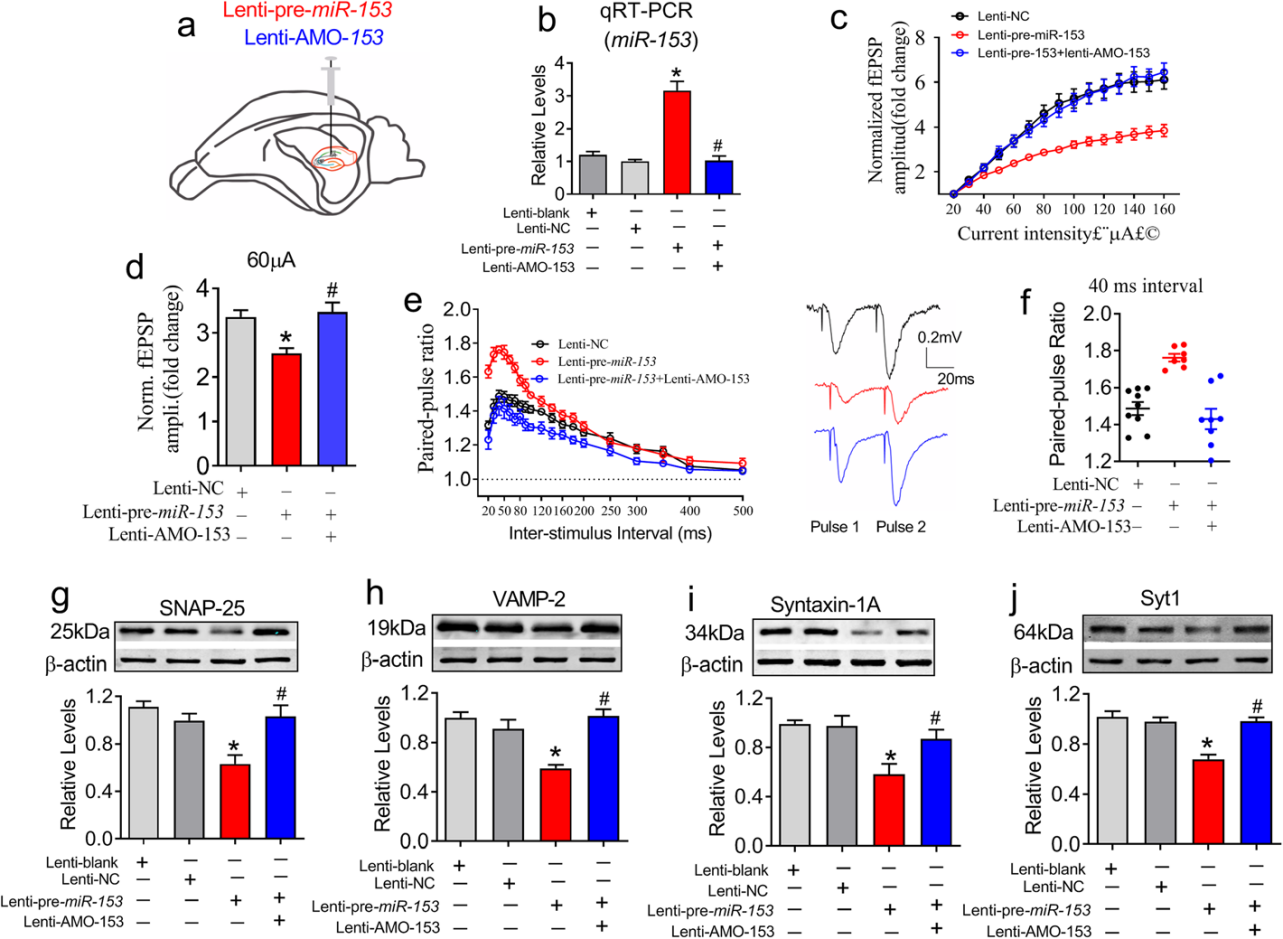
*MiR-153* gain-of-function impairs presynaptic vesicle release in rat hippocampi. **a** Schematic diagram of stereotactic lentivirus injection into the CA1 region of the hippocampus. **b** Relative levels of *miR-153* in the hippocampi of rats (*P*_Levene_ = 0.069, one-way ANOVA: F = 39.14, *P* < 0.0001). *n* = 6. **c** Lenti-AMO-153 reversed the repression effect of lenti-pre-*miR-153* on fEPSP in the CA3-CA1 pathway. *χ*^*2*^_Mauchly_ = 484.594, *P* < 0.0001; F_total (14, 295)_ = 12.618, *P* < 0.0001, *n* = 7 for lenti-pre-*miR-153* group, *n* = 8 for the lenti-AMO-153 and sham groups. **d** The fold change in the fEPSP amplitude following 60 μA current stimulation (*P*_Levene_ = 0.620, one-way ANOVA: F = 3.993, *P* = 0.032). **e** Lenti-AMO-153 reversed the increased in the PPR induced by lenti-pre-*miR-153*. *χ*^*2*^_Mauchly_ = 509.893, F_(18, 433)_ = 18.968, *P* < 0.0001. Insertion: representive of EPSP traces. **f** Scatter dot plot showing the increased PPR with a 40 ms ISI in the lenti-pre-*miR-153* injected rats compared with that in the lenti-NC-injected rats, and this increase in PPR was prevented by the coinjection with lenti-AMO-153 (*P*_Levene_ = 0.186, one-way ANOVA: F = 17.454, *P* < 0.0001). **g**-**j** Lenti-pre-*miR-153* repressed the protein levels of SNAP-25 (*P*_Levene_ = 0.11, one-way ANOVA: F = 9.021, *P* = 0.001), VAMP-2 (*P*_Levene_ = 0.352, one-way ANOVA: F = 13.824, *P* < 0.0001), syntaxin-1A (*P*_Levene_ = 0.193, one-way ANOVA: F = 7.023, *P* = 0.002) and Syt1 (*P*_Levene_ = 0.871, one-way ANOVA: F = 17.301, *P* < 0.0001) in the hippocampal synaptosomes. n = 6. *P < 0.05 vs lenti-NC; ^#^*P* < 0.05 vs lenti-pre-*miR-153*

**Fig. 6:**
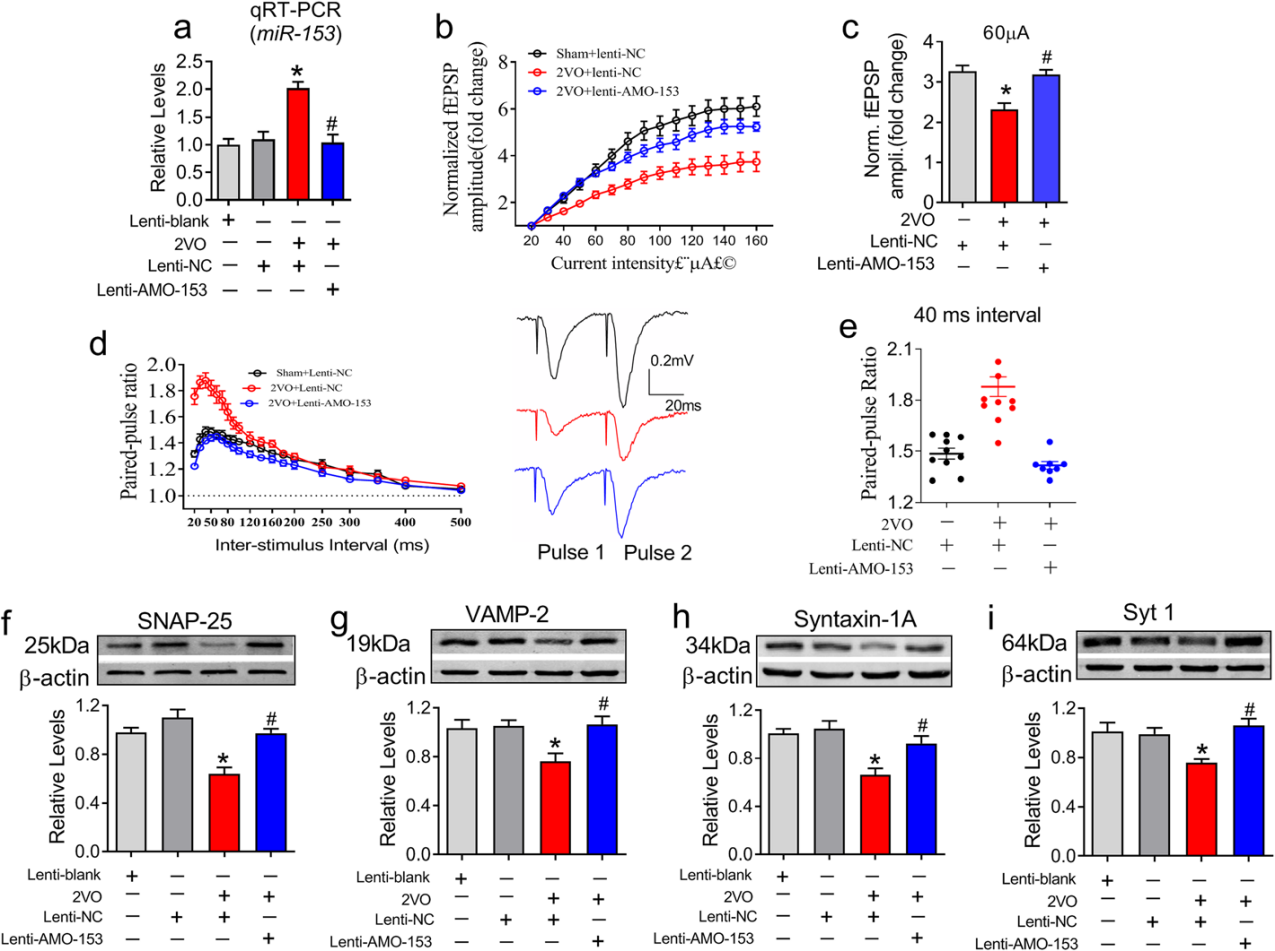
Knockdown of *miR-153* attenuates the decline in hippocampal presynaptic plasticity in 2VO rats. **a** Lenti-AMO-153 injection inhibited *miR-153* expression in the hippocampi of 2VO rats (*P*_Levene_ = 0.58, one-way ANOVA: F = 10.301, *P* = 0.0001). n = 6. **b** Lenti-AMO-153 attenuated the repression of fEPSP recorded in 2VO rats. *χ*^*2*^_Mauchly_ = 549.258, *P* < 0.0001; F_total (14, 309)_ = 11.229, *P* < 0.0001. **c** Bar graph showing the fold changes in the fEPSP amplitude under 60 μA current stimulation (*P*_Levene_ = 0.598, one-way ANOVA: F = 8.114, *P* = 0.002). *n* = 10. **d** Lenti-AMO-153 reversed the increase in the PPR in 2VO rats. Right insertion: sample of PPR fEPSP traces. *χ*^*2*^_Mauchly_ = 596.166, P < 0.0001; F_(18, 505)_ = 20.561, *P* < 0.0001. n = 10 for the 2VO group, *n* = 9 for the lenti-NCgroup, n = 8 for the lenti-AMO-153 group. **e** Lenti-AMO-153 prevented the increase in the PPR in 2VO rats (*P*_Levene_ = 0.001, one-way ANOVA: F = 31.707, *P* < 0.0001). **f**-**i** Lenti-AMO*-153* derepressed the protein levels of SNAP-25 (*P*_Levene_ = 0.324, one-way ANOVA: F = 15.658, *P* < 0.0001), VAMP-2 (*P*_Levene_ = 0.752, one-way ANOVA: F = 5.288, *P* = 0.01), syntaxin-1A (*P*_Levene_ = 0.817, one-way ANOVA: F = 9.248, *P* < 0.0001) and Syt1 (*P*_Levene_ = 0.246, one-way ANOVA: F = 6.3.60, *P* = 0.003) in hippocampal synaptosomes in 2VO rats. Fisher’s PLSD test was used for the post hoc analyses of the two-group comparisons. n = 6, *P < 0.05 vs Lenti-NC; ^#^*P* < 0.05 vs 2VO

Subsequently, we investigated whether the upregulation of *miR-153* affect vesicle release in vivo. We designed three lentiviral constructs, named lenti-mis-pre-*miR-153* as NC (lenti-NC), lenti-pre-*miR-153* and lenti-AMO-153, and stereotaxically injected them directly into the bilateral hippocampal CA1 subfields of each rat [3] (Fig. 5a and b, *P* < 0.0001). Relative to lenti-NC injection, lenti-pre-*miR-153* application resulted in an overall decrease in the fEPSP responsiveness in the CA1 area and more quickly saturated electrical stimulation as indicated by the I-O curve (Fig. 5c, *P* < 0.0001 and Fig. 6d, *P* = 0.032), which was similar to that of the 2VO rat (Fig. 1c). Similarly, lenti-pre-*miR-153* significantly elevated the synaptic PPR when the ISI was between 20 ms and 100 ms, and this increase was prevented by coinjection with lenti-AMO-153 (Fig. 5e and f, *P* < 0.0001). Accordingly, we observed that lenti-pre-*miR-153* significantly inhibited the protein expression of SNAP-25 (*P* = 0.001), VAMP-2 (*P* < 0.0001), syntaxin-1A (*P* = 0.002) and Syt1 (*P* < 0.0001) in the rat hippocampi (Fig. 5g - j), and these effects were reversed by lenti-AMO-153 treatment. These results indicate that *miR-153* gain-of-function disturbs hippocampal vesicle release by inhibiting vesicle-related proteins in vivo.

### Knockdown of *miR-153* rescues hippocampal presynaptic vesicle release decline in 2VO rats

Because 2VO elicited increased *miR-153* levels in the hippocampus of 2VO rats (Fig. 6a), we next sought to determine whether knockdown of *miR-153* could improve the decreased presynaptic vesicle release in 2VO rats. As predicted, compared with lenti-NC, lenti-AMO-153 significantly inhibited the high expression of *miR-153* in the hippocampi of 2VO rats (Fig. 6a, *P* = 0.0001). Moreover, lenti-AMO-153 significantly rescued the decreased fEPSP amplitude (Fig. 6b, *P* < 0.0001 and c, *P* = 0.002) and impaired synaptic facilitation (Fig. 6d and e, *P* < 0.0001) in 2VO rats. Correspondingly, lenti-AMO-153 successfully reversed the decreased expression of SNAP-25 (*P* < 0.0001), VAMP-2 (*P* = 0.01), syntaxin-1A (*P* < 0.0001) and Syt1 (*P* = 0.003) in the hippocampi of 2VO rats (Fig. 6f-i). These results suggest that lenti-AMO-153 reversed not only the release probability but also the expression of vesicle release-related proteins in CBH rats.

### Knockdown of *miR-153* attenuates cognitive decline of 2VO rats

**Fig. 7:**
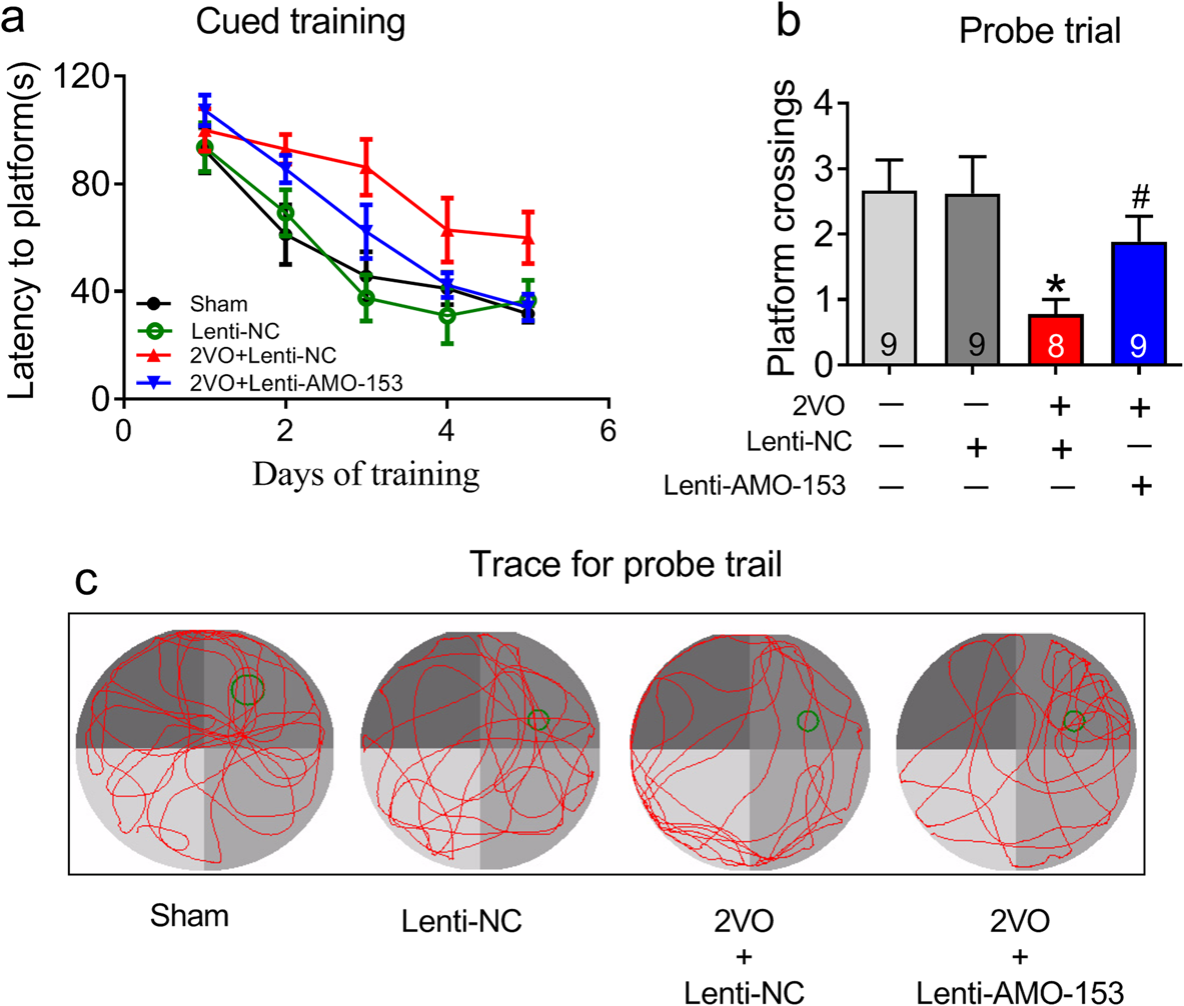
Knockdown of *miR-153* attenuates learning and memory deficits in the 2VO model. **a** Knockdown of *miR-153* by injecting lenti-AMO-153 reversed the increase in the daily average escape latency to locate the platform from three nontarget quadrants in 2VO rats. *χ*^*2*^_Mauchly_ = 16.20, *P* = 0.063;; F_total (4, 104)_ = 7.404, *P* = 0.001. **b** Average number of platform crossings during the probe trial. *P*_Levene_ = 0.005, one-way ANOVA: F = 4.736, *P* = 0.008. n = 8, *P < 0.05 vs lenti-NC; ^#^*P* < 0.05 vs 2VO + lenti-AMO-153. One-way ANOVA, Fisher’s PLSD test were used for the post hoc analyses of the two-group comparisons. **c** Represent path tracing of the probe trial test on day 6 in the MWM test for each group

Subsequently, we investigated whether *miR-153* knockdown could prevent the dementia phenotype induced by 2VO. As expected, compared with age-matched 2VO rats that were transfected with lenti-NC, 2VO rats transfected with lenti-AMO-153 exhibited a significantly shortened latency to arrive at the platform (Fig. 7a, *P* = 0.001). In the probe trial, lenti-AMO-153 treatment increased the number of platform crossings in 2VO rats (Fig. 7b and c, *P* = 0.08). These results imply that knockdown of *miR-153* improved spatial memory in 2VO rats.

## Discussion

We are the first to report that CBH impairs presynaptic plasticity by disturbing synaptic vesicle release. The disturbed process was due to a blockade of presynaptic vesicle fusion with the presynaptic membrane controlled by *miR-153*, which posttranscriptionally repressed the expression of fusion-related proteins, including the SNARE-complex proteins SNAP-25, VAMP-2 and syntaxin-1A and the Ca^2+^ sensor protein Syt1 by targeting the 3’UTRs of the *Snap25*, *VAMP2*, *Stx1a* and *Syt1* genes (Fig. S4). This mechanism not only deepens our understanding of CBH-induced brain dysfunction, but also leads to a new preventive or treatment strategy for AD or VaD.

The number of formed SNARE complexes is widely known to be the rate-limiting index controlling the quantity and speed of presynaptic vesicle release. Although 2 ~ 3 SNARE-complexes are sufficient to mediate the fusion process between vesicles and presynaptic membranes, more SNARE complexes trigger greater vesicle release [38]. Here, CBH downregulated the expression of SNAP-25, VAMP-2 and syntaxin-1A, suggesting that CBH induced vesicle release impairment may reduce the number of SNARE complexes. Synaptic vesicle fusion is not only mediated by SNARE proteins but also co-regulated by SM proteins that control fast vesicle release by opening the fusion-pore by engaging in the formation of the *trans*-SNARE/SM-complex [15, 39]. However, in our study, Munc-18-1 expression was unchanged in the hippocampi of 2VO rats, suggesting that the CBH-induced impairment in synaptic fusion is associated with reduced SNARE protein expression but not SM protein expression. Furthermore, prior to fusion-pore opening, synaptic vesicles need to be docked and primed by triggering the formation of the *trans*-SNARE/SM complex, which is mediated by rapid Ca^2+^ binding to the Ca^2+^-sensor protein Syt1 [40, 41]. Interestingly, the protein level of Syt1 was significantly reduced in the rat hippocampi following CBH; however, the level of its co-factor complexin1/2 was unchanged. Thus, the above data provide profound evidence that CBH impairs presynaptic plasticity through effects on vesicle fusion-related proteins.

A previous study reported that *miR-153* is a contextual fear-induced miRNA and affects fear memory by targeting the *Snap25* and *Vamp2* genes [23]. Here, we first reported that *miR-153* is also a CBH-induced miRNA. In addition, we found that increased *miR-153* levels in the plasma of VaD patients, suggesting that *miR-153* is a potential biomarker for the preclinical prediction of VaD.

Surprisingly, bioinformatics analysis, siRNA and miR-masking experiments revealed that all the vesicle fusion-related proteins with altered expression in the hippocampi of CBH rats, including SNAP-25, VAMP-2, syntaxin-1A and Syt1, are *miR-153* targets. Notably, the mRNA level of *Snap25* was significantly increased in the hippocampi of CBH rats, whereas the mRNA level of *VAMP2*, *Stx1a* and *Syt1* remained unchanged; however, the protein level of SNAP-25 was decreased in the same with manner as that VAMP-2, syntaxin-1A and Syt1. This phenomenon suggests that the inhibitory effect of *miR-153* on SNAP-25 was the strongest among these proteins, which was further proven by the luciferase reporter gene expression assay. To clarify the involvement of *miR-153* in the CBH-induced presynaptic vesicle release impairment, we performed both in vitro and in vivo experiments. First, using the FM1–43 staining method, we demonstrated that *miR-153* gain-of-function markedly blocked KCl-induced vesicle release in NRNs, and this effect of *miR-153* was completely prevented by the cotransfection of the antisense AMO-153. Subsequently, by performing four gene-specific miR-masks, we found that the contributions of these four proteins to the *miR-153*-induced impairment in vesicle release were different. For example, *Vamp2-ODN* and *Snap25-ODN* almost completely blocked the *miR-153*- induced reduction in vesicle release within 5 s after KCl stimulation, having the same effect of all four ODNs combined. At 10 s after KCl application, the degree of the rescue effect (from strong to weak) of each individual ODN was *Vamp2-ODN > Snap25-ODN > Syt1-ODN > Stx1a-ODN*. Interestingly, as time extended to 40 s after KCl stimulation, the effect of *Stx1a-ODN* and *Syt1-ODN* gradually improved and reached to a level similar to that of *Vamp2-ODN*. These results indicate that VAMP-2 and/or SNAP-25 are the rate-limiting proteins under CBH status. Second, as predicted, in vivo, application of lenti-pre-*miR-153* decreased fEPSP amplitude and increased the PPR in the CA3-CA1 pathway and reduced the expression of the vesicle-related proteins similar to the observed effects of 2VO surgery. Importantly, all these changes were prevented by lenti-AMO-153 treatment. Hence, these data provide pronounced evidence that *miR-153* is involved in the impaired vesicle release process in 2VO rats by posttranscriptionally regulating the expression of a battery of vesicle-related proteins.

Previous studies have reported that CBH induces a series of pathological changes involving Aβ aggregation, Tau hyperphosphorylation, dendritic remodelling and cell death through the single microRNA- *miR-195*, which targets a set of genes associated with the Aβ cascade response, including *APP* (encoding amyloid precursor protein, APP) [3], *BACE1* (encoding β secretases, BACE1) [3], *cdk5r1* (encoding p35) [4], *Ppme1* (encoding methylesterase 1, PME-1) [42] and *tnfrsf21* (encoding death receptor 6, DR6) [5]. Importantly, application of *miR-195* by stereotaxic injection of lenti-pre-*miR-195* into the hippocampus could partially reduce dementia-vulnerability triggered by 2VO [3].

In this study, we found that the single microRNA- *miR-153* can regulate a battery of coordinating molecules that control presynaptic vesicle release. Interestingly, knockdown of *miR-153* by lenti-AMO-153 also partially attenuated 2VO-induced dementia-vulnerability. These observations suggest that a combination of *miR-195* upregulation and *miR-153* downregualtion may be the best strategy to prevent or treat cognitive decline induced by CBH.

In the present study, although we demonstrated the direct action of *miR-153* on presynaptic vesicle release by its targeting of multiple vesicle release-related proteins, we did not investigate the mechanism by which CBH upregulates of *miR-153*. A previous study reported that hypoxia-stimulated endoplasmic reticulum stress promotes *miR-153* transcription by activating IRE1α and its downstream transcription factor X-box binding protein 1 (XBP1). XBP1 directly binds to the promoter of the *miR-153* host gene PTPRN [43]. Whether XBP1 alone or together with other elements involves in CBH induced upregulation of *miR-153* needs to be identified further.

## Conclusion

Overexpression of *miR-153* controls CBH induced presynaptic vesicle release impairment by post-transcriptionally regulating the expression of four vesicle release-related proteins through targeting the 3’UTRs of the *Stx1a*, *Snap25*, *Vamp2* and *Syt1* genes (Fig. S4). These findings provide new insights into the molecular mechanism of CBH-induced presynaptic plasticity impairment at the miRNA level. Our findings suggest that *miR-153* might be a candidate for dementia-targeting gene-therapy in the future.

## Supplementary information

**Additional file 1.**

## Data Availability

The data used in this study are available from the corresponding author upon reasonable request.

## Abbreviations

AD: Alzheimer’s disease
VaD: vascular dementia
CBH: chronic brain hypoperfusion
2VO: bilateral common carotid artery ligation
fEPSPs: field excitatory postsynaptic potentials
PPF: paired-pulse facilitation
SNAREs: soluble N-ethylmaleimide-sensitive factor attachment receptor proteins
VAMP-2: vesicle associated membrane protein 2
SNAP-25: synaptosomal-associated protein 25
Syt1: synaptotagmin-1
NRNs: neonatal rat hippocampal and cortical neurons
ODNs: oligodeoxynucleotides
MMSE: Mini-Mental State Examination
MoCA: Montreal Cognitive Assessment
HAMD: Hamilton Depression Scale
TTC: 2,3,5-triphenyltetrazolium chloride
MWM: morris water maze
PPR: paired-pulse ratio
CBF: cerebral blood flow
PBS: phosphate-buffered saline
ISI: interstimulus intervals
