# Supplementary material for: MicroRNA-153 impairs presynaptic plasticity by blocking vesicle release following chronic brain hypoperfusion
Source: Cell Commun Signal. 2020 Apr 6;18:57. doi: 10.1186/s12964-020-00551-8 (PMC7137307; doi:10.1186/s12964-020-00551-8)
Supplement: Supplementary file 2 — Additional file 1. [file 12964_2020_551_MOESM2_ESM.docx]

**MicroRNA-153 impairs presynaptic plasticity by blocking vesicle release following chronic brain hypoperfusion.**

Mei-Ling Yan^†^, Shuai Zhang^†^, Hong-Mei Zhao, Sheng-Nan Xia, Zhuo Jin, Yi Xu, Lin Yang, Yang Qu, Si-Yu Huang, Ming-Jing Duan, Meng Mao, Xiao-Bin An, Chandan Mishra, Xin-Yu Zhang, Li-Hua Sun, Jing Ai*

Department of Pharmacology (The State-Province Key Laboratories of Biomedicine-Pharmaceutics of China), College of Pharmacy of Harbin Medical University, Harbin, Heilongjiang Province, China, 150086

^†^Mei-Ling Yan and Shuai Zhang contributed equally to this work.

*Correspondence to Jing Ai: [azhrbmu@126.com](mailto:azhrbmu@126.com) or aijing@ems.hrbmu.edu.cn

Department of Pharmacology (The State-Province Key Laboratories of Biomedicine-Pharmaceutics of China), College of Pharmacy of Harbin Medical University, Harbin, Heilongjiang Province, China, 150086; Tel.: +86 451 86671354; Fax: +86 451 86671354.

**Additional file 1:** Full length 3’UTR and mutant 3’UTR of *Snap25, Vamp2, Stx1a* and*, Syt1* genes containing the putative binding sequences for *miR-153*.

*Snap25-3’UTR:* (the binding site of *miR-153* is marked by yellow color)

atctgcccttctgctgtgctctcctccaatgttgttggacaagagagaagagagctccttcatgcttctctcatggtattacctagtaagacttacacacacacacacacacacacacacacacacacacacacacacacacagagtcagtcacccccattgtaaatgtcctgtgtggtttgtcagcttcccaatgataccatgtgtcttttgttttctccggctctctttctttccaaaggttgtacatagtggtcatctggtgactctatttcctgacttaagatgtcttgggtctctctctttttcctttttcttttctcagtggcgtttgctgaatgacaacaatttaggaatgctcaatgtactgttgatttttctcaatacacagtattgttcttgtaaaactgtgacattccacagagctactaccacagtcctttcttagggtgtcaggctctgaatctctccaaatgtgctgtctttggttcctcatggctattctttgtctttatgatttcataattagacaatgtgaaattacataacaggcattgcactaaaagtgatgtgatttatgcatttatgcatgagaactaaatagacttttagattcctacttaaacaaaaactttccatgacagtagcatactgacaagaaaacacacacaacagcaacaataacaaagcaacaactacgcatgctcagtattgggacactgtcaagattaagtcataccagcaaaacctgcagctgtgtcaccttcttctgtcaacatacagactgatcataatcatcccttctttacacacacacacacacacacacacacacacacacacacacacacaaatggatttaacacaccattaatcatttctagcaaaatatatgtttggctgaaactatgtgaaatgggtgtaatatagggtttgtcgaatgcttttgaaagctctgttttggagacaatactcttgtggaaaacgtgaagatcttctaagtctggctcttgttgatcaccatttccctgtggtttgtcatcagtacaactctttgttgcttaatctagagctatgcacaccaaattgctgagatgtttagtagctgataaagaaacctttaaaaaattatataaatgaatgaaatatagataaactgtgagataaatatcattacagcatgtatattaaatccctcctgtctcctctgttggtttgtgaagtgatttgacattttgtagctagtttaaaattattaaaaattatagatcccaa

*mut Snap25-3’UTR:* (the mutated DNA sequences of *miR-153* binding site is marked by red color) atctgcccttctgctgtgctctcctccaatgttgttggacaagagagaagagagctccttcatgcttctctcatggtattacctagtaagacttacacacacacacacacacacacacacacacacacacacacacacacacagagtcagtcacccccattgtaaatgtcctgtgtggtttgtcagcttcccaatgataccatgtgtcttttgttttctccggctctctttctttccaaaggttgtacatagtggtcatctggtgactctatttcctgacttaagatgtcttgggtctctctctttttcctttttcttttctcagtggcgtttgctgaatgacaacaatttaggaatgctcaatgtactgttgatttttctcaatacacagtattgttcttgtaaaactgtgacattccacagagctactaccacagtcctttcttagggtgtcaggctctgaatctctccaaatgtgctgtctttggttcctcatggctattctttgtctttatgatttcataattagacaatgtgaaattacataacaggcattgcactaaaagtgatgtgatttatgcatttatgcatgagaactaaatagacttttagattcctacttaaacaaaaactttccatgacagtagcatactgacaagaaaacacacacaacagcaacaataacaaagcaacaactacgcatgctcagtattgggacactgtcaagattaagtcataccagcaaaacctgcagctgtgtcaccttcttctgtcaacatacagactgatcataatcatcccttctttacacacacacacacacacacacacacacacacacacacacacacaaatggatttaacacaccattaatcatttctagcaaaatatatgtttggctgaaactatgtgaaatgggtgtaatatagggtttgtcgaatgcttttgaaagctctgttttggagacaatactcttgtggaaaacgtgaagatcttctaagtctggctcttgttgatcaccatttccctgtggtttgtcatcagtacaactctttgttgcttaatctagagtcgcatgcaccaaattgctgagatgtttagtagctgataaagaaacctttaaaaaattatataaatgaatgaaatatagataaactgtgagataaatatcattacagcatgtatattaaatccctcctgtctcctctgttggtttgtgaagtgatttgacattttgtagctagtttaaaattattaaaaattatagatcccaa

*Vamp2-3’UTR:* (the binding site of *miR-153* is marked by yellow color)

ggttgtccttaacaccccttcctgaggttcccatcacctctctctctcaccctctgccccctttcctctttcctgttcagccccaagtcccttcatttgcatctgctatgcaatagcccctctcctccttcccttggatttaaccaatccttcccctatcttccctgtacaattccagaccctccccaaacagagcaaaccccacagaaacaaacaaaacaccccacctgtctaggcttccataggttgcatctttgtatcccttgggagcctctaagactggtcctacttggtcctaagaatcccaaggttttctgggagcttccaacatgttgattagcatatcatttgcatacactgtcttttttttttccctgggtttcttcctttctgttccattactcttcactcttctgtgttttttttttttctttctttcttttcggaagagttagtttccattggtcctcttatcacacttttatttgcacgacattatctgcaggtggtggggagcctgggcttttggggaactaggaccccaaagttgcctcctcccagcccctctagtctagtttgcttcccttaccccattttccacacctcttgtaccctcctctccctcccccagctggtgtgtaagtgtcttggagttcagtgtgttatgatggaccaataattctgccacttggggtctctccctacattcctgctccccagttttcgtgtggggcattcagctgacattcccgggggtctccctccctcccatctgcctgatctgctgcctcctcccaccaggagaactggaggctgaccacaatctggttctttgaggaggggtggctctagtgtgtgtgggggtcatcactgccttggggaggagtgggacaaaacagaaccccccctaattcctgcctgaaatctctggcctcatccttgctagaggttggactgaaaactttcctccccaatcctgggggggggtattaccccccatcactgcccagctcctctgactgcccccctgtattcagggtgggggtactagtcactgccaatatgtgtatgggacttgctggaggatggggatccttgtccttctctagggctgctgagccctgagagagagagagagagagagagagagagagagagagagagagagagagagagggaagcaagctatttccctgttgggtacaatgatagagaaaggatctaatctttaaaaatggcacagttttaggggtctgagggtacagccccttaacctgcctcttggggggggtgttccccaaactcttcccccacacacaccaggttttctgtgtggaggggaaccaaggagatctaaactgtggtgtgaaagggtaggagagatgctgggggtgggggtgcttgtgttttagacccccaatattatcccagtgtcccctgccttccttcttcccctgccccatgcccccaattctgtggcgcatccagattgtgaaaatgtacaataaacgtgtaatgagtaaa

*mut Vamp2 -3’UTR:* (the mutated DNA sequences of *miR-153* binding site is marked by red color)

ggttgtccttaacaccccttcctgaggttcccatcacctctctctctcaccctctgccccctttcctctttcctgttcagccccaagtcccttcatttgcatctggatacgtatagcccctctcctccttcccttggatttaaccaatccttcccctatcttccctgtacaattccagaccctccccaaacagagcaaaccccacagaaacaaacaaaacaccccacctgtctaggcttccataggttgcatctttgtatcccttgggagcctctaagactggtcctacttggtcctaagaatcccaaggttttctgggagcttccaacatgttgattagcatatcatttgcatacactgtcttttttttttccctgggtttcttcctttctgttccattactcttcactcttctgtgttttttttttttctttctttcttttcggaagagttagtttccattggtcctcttatcacacttttatttgcacgacattatctgcaggtggtggggagcctgggcttttggggaactaggaccccaaagttgcctcctcccagcccctctagtctagtttgcttcccttaccccattttccacacctcttgtaccctcctctccctcccccagctggtgtgtaagtgtcttggagttcagtgtgttatgatggaccaataattctgccacttggggtctctccctacattcctgctccccagttttcgtgtggggcattcagctgacattcccgggggtctccctccctcccatctgcctgatctgctgcctcctcccaccaggagaactggaggctgaccacaatctggttctttgaggaggggtggc tctagtgtgtgtgggggtcatcactgccttggggaggagtgggacaaaacagaaccccccctaattcctgcctgaaatctctggcctcatccttgctagaggttggactgaaaactttcctccccaatcctgggggggggtattaccccccatcactgcccagctcctctgactgcccccctgtattcagggtgggggtactagtcactgccaatatgtgtatgggacttgctggaggatggggatccttgtccttctctagggctgctgagccctgagagagagagagagagagagagagagagagagagagagagagagagagagagggaagcaagctatttccctgttgggtacaatgatagagaaaggatctaatctttaaaaatggcacagttttaggggtctgagggtacagccccttaacctgcctcttggggggggtgttccccaaactcttcccccacacacaccaggttttctgtgtggaggggaaccaaggagatctaaactgtggtgtgaaagggtaggagagatgctgggggtgggggtgcttgtgttttagacccccaatattatcccagtgtcccctgccttccttcttcccctgccccatgcccccaattctgtggcgcatccagattgtgaaaatgtacaataaacgtgtaatgagtaaa

*Stx1a-3’UTR:* (the binding site of *miR-153* is marked by yellow color)

Aaaccaccccacccacggctccattctggatgggtctccctgaggaggcccctggctgctgcacctagctgggttgccctccccactcctgccttctggctgggagtccttttccctcccatccaacaccgctccctctctgccatgaggctcccgtccccaccaccctgccccaagccgtgtcgtgtgcatgatcttgtgacagtgtgtgtctgtacaggaggcagaggggagcaggatcgggaacagccagaggggctgggtacaggccagtgtgggcaagactcgggccctggccaggtccgccttccttcaggcctggggcctacgcttccctgggactcaggctcctttctggaccccaaccttgccctcactcgccctgccctctggcttcctcagctctccccaccatgccaaggcacctggagggtggggaccagctggtcacatggtgctgcttttcaggttaggggtggggacagctcagcactgagtctttgttagctgcccactgccaggatgctcagggtgccacggctgctggtgtgctaggagcacccagtacccctctttggcaaagcctgacagtgtctctggcctcagctgcccttaccacagccctgggagtcctgttcctgggctgggcctgagcctagtgatcctttgctaagaagctcagtggtgccatctccagccttgctctgagctggagaggtggagcaggccatagtcctctgcccacagtctctaacgggcatgttaagtcgtggccggagttgcatgttagggacagcggttccctgctccctttctgctctgaaaagccaggtgtcactttgggcctgcagtctcaccctgccctgtctcccattgatgtgccacgtggtgtcaggtgtactggatgcagtattcagcagccagctggggagggggctccccactttcctcccctgccaacttggggcttctcagagtcaaaaatgtacccccatgccccaggacccctttctcatccacaggcaaggagtatgcatgcgactgcatgcagcgggagctggggccgtgtctgtgtgccccttcccctccgctttgctcctgcccagtgactgaccactgtccgtgctgccttctctcatggccacttccctttaccccgtcaccaaaggtctcggtacaaccagctgcccattttgtgagatttttatgtagaataaacatttgtatctgga

*mutStx1a-3’UTR:* (the mutated DNA sequences of *miR-153* binding site is marked by red color)

Aaaccaccccacccacggctccattctggatgggtctccctgaggaggcccctggctgctgcacctagctgggttgccctccccactcctgccttctggctgggagtccttttccctcccatccaacaccgctccctctctgccatgaggctcccgtccccaccaccctgccccaagccgtgtcgtgtgcatgatcttgtgacagtgtgtgtctgtacaggaggcagaggggagcaggatcgggaacagccagaggggctgggtacaggccagtgtgggcaagactcgggccctggccaggtccgccttccttcaggcctggggcctacgcttccctgggactcaggctcctttctggaccccaaccttgccctcactcgccctgccctctggcttcctcagctctccccaccatgccaaggcacctggagggtggggaccagctggtcacatggtgctgcttttcaggttaggggtggggacagctcagcactgagtctttgttagctgcccactgccaggatgctcagggtgccacggctgctggtgtgctaggagcacccagtacccctctttggcaaagcctgacagtgtctctggcctcagctgcccttaccacagccctgggagtcctgttcctgggctgggcctgagcctagtgatcctttgctaagaagctcagtggtgccatctccagccttgctctgagctggagaggtggagcaggccatagtcctctgcccacagtctctaacgggcatgttaagtcgtggccggagttgcatgttagggacagcggttccctgctccctttctgctctgaaaagccaggtgtcactttgttaagtacttctcaccctgccctgtctcccattgatgtgccacgtggtgtcaggtgtactggatgcagtattcagcagccagctggggagggggctccccactttcctcccctgccaacttggggcttctcagagtcaaaaatgtacccccatgccccaggacccctttctcatccacaggcaaggagtatgcatgcgactgcatgcagcgggagctggggccgtgtctgtgtgccccttcccctccgctttgctcctgcccagtgactgaccactgtccgtgctgccttctctcatggccacttccctttaccccgtcaccaaaggtctcggtacaaccagctgcccattttgtgagatttttatgtagaataaacatttgtatctgga

*Syt1-3’UTR:* (the binding site of *miR-153* is marked by yellow color) agggaaaacgaagcctttctgcatctgcccacatagtgctctttagccagtatctgtaaatacctcagtaatatgggtccttttggtttccagccatgcattcctgatacaatccagtggtacttcaaatcctgttttaatttgcacaaatttaagtgtagaaagcccttatgccctccatcataccactgccctccaaatctactcttcttttaagcaatatgatgtgtagatagagcatgactgaaattatgtattgtatcacactgttgtatataccagtatgctaaagatttatttctagtttgtgtatttgtatgttgtaagcgtttcctaacctgtgtatatctagatgtttttaataagatgttctattttaaactatgtaaattgactgagatataggagagctgataatatattatatggtaaatatagtatcgtctgcattccagcaaaaatatcaacttgaaaggcactagtacagttaaaccaacatcttaaaggacaacttaaacctgagctttctattgaatcctttgagtaccaagattcgctcacacaacacctttgatgggcgaacccaattttgtagaattctttcacaggcaaatagcatgacctgagcagcatctgggctgacctcaaggaagcaaagccacaaaccagaatagcatctgtctgtctgtacctgcaaagccaaagccatggcttcgctcttacagtcaaggaagcaatgaacaggagccaatgcgttcctaccactgcatctagcatagcttcatggtggtgttctctgtgtgtgcgtgtgcaagcgtgaaagtgtatgcacgtgtgtatgtgtggtgcatgcctttgtttggggttagggtgggggaggaggagctgagggaagtcagcgtttctgaaatattgcctgcctgtttaaacagaaaattatagctctccattgtcacatttatataaaacgtgcaacctgggaattctgatccggatttcaccccaatattgattccaaaaggtatgcgcgtgagactttgtaacaaaatattttattatacaaaaccagattagaaggaatgcagaatatttttaacgcagcaatctgtgcttattccacaaagtgactttgtggtaaacagacagtattgtaaccccacgaaaagacggaatataacagttagccatagttctgaatgcacttcgacgaagccaaaacagacagctagtgatctttttatatgctctttttacggagttttaatttgtcctttaaacaaaggtgaaacaaaccaagaacaagttctcgcaaactgaagcaacctcttatgtacactagatgcttgacttaggaggagtttttaaatgttctcaatgttattctgtagtaaatggcactattatgaagccactagtcattccatatgagtcttaaggactgctctgtgtaacactgtgactgccccgtgtgcttagacacgtagtttcctcagtggatagcactcaacttactccgtagtgatattgtaacaatactgccattccctcttactgcactgcccaacatgtgtgtagcacaaacagttctcattcctaaggaccaattcagaactgaacagctatgcataggacagaaagatacatagaccgggtgtgggagaacacacagcattttgtcaacactgtgcactagtcacatttgtcctgctgccggtagacagccacttcaggaagtgagcctgctacctaacaccgcttctagactcttctcccacttgctattgtggcccgttttcacctccaggtcacagagaatggcaacatcctgaagggagagaccatcttcacatctaccaaaataaaatggaggaatgctaagcatggcctcgtgcttgatctttaggaattagctccgtgttttggacaaaactcaagagaatccccaatagggctggtggtagactttaagcacggggtcggctgctcctcctgcacacacaacacaaaagctaacccctggttgtgattcttccctcatgagagaagaggcaaaccctttgcccttcactcccatcacagcaaactttcagacctagaacagacacacaggacaaggagcaaatccttccctatggatgaacagcacgtttccaacattaaaaccacagatgataggaaacacatactcataggtgagattaaacagcagtttaaacaggagactcaaatgaggggctttcctatctaagggatcaagtcctaccaaagagaaggaacaccttaaataccagacactgacatttaatttcatcatctcccgacttgagttgtacacaatggaacatttccgaggacgcagctccgagctgccgaactgacattacttcctgcattacaatgatactagcacattctcttgcaacactgccaacatgggattgtcaccatagagttagttggtactatatcattctcttgtgagccggtgactggacctgctttctgaccaagatccatcctctgataagccacatgtacctttctgacaatgcagtgtgaagtcttagaagctgatgccctagaaagatcctagttgcctttgtgtatacttactgcctgcttgagtgtttctatgtgtggattttctctgtgtctcgtagaaatgttggggtgttttcttctgccataaggcttgtgacccgcgagccaattcccttagctgtactttcccttcattttttgataagtggtttaaattctgtttcactttgtgtagtgaaccccatggtagttttctgattgttgttaaaaaaaatgacttaacatattacatggacactcaataaaaatgttttatttcctgttga;

*mutSyt1 3’ UTR:* (the mutated DNA sequences of *miR-153* binding site is marked by red color)

agggaaaacgaagcctttctgcatctgcccacatagtgctctttagccagtatctgtaaatacctcagtaatatgggtccttttggtttccagccatgcattcctgatacaatccagtggtacttcaaatcctgttttaatttgcacaaatttaagtgtagaaagcccttatgccctccatcataccactgccctccaaatctactcttcttttaagcaatatgatgtgtagatagagcatgactgaaattatgtattgtatcacactgttgtatataccagtatgctaaagatttatttctagtttgtgtatttgtatgttgtaagcgtttcctaacctgtgtatatctagatgtttttaataagatgttctattttaaactatgtaaattgactgagatataggagagctgataatatattatatggtaaatatagtatcgtctgcattccagcaaaaatatcaacttgaaaggcactagtacagttaaaccaacatcttaaaggacaacttaaacctgagctttctattgaatcctttgagtaccaagattcgctcacacaacacctttgatgggcgaacccaattttgtagaattctttcacaggcaaatagcatgacctgagcagcatctgggctgacctcaaggaagcaaagccacaaaccagaatagcatctgtctgtctgtacctgcaaagccaaagccatggcttcgctcttacagtcaaggaagcaatgaacaggagccaatgcgttcctaccactgcatctagcatagcttcatggtggtgttctctgtgtgtgcgtgtgcaagcgtgaaagtgtatgcacgtgtgtatgtgtggtgcatgcctttgtttggggttagggtgggggaggaggagctgagggaagtcagcgtttctgaaatattgcctgcctgtttaaacagaaaattatagctctccattgtcacatttatataaaacgtgcaacctgggaattctgatccggatttcaccccaatattgattccaaaaggtatgcgcgtgagactttgtaacaaaatattttattatacaaaaccagattagaaggaatgcagaatatttttaacgcagcaatctgtgcttattccacaaagtgactttgtggtaaacagacagtattgtaaccccacgaaaagacggaatataacagttagccatagttctgaatgcacttcgacgaagccaaaacagacagctagtgatctttttatatgctctttttacggagttttaatttgtcctttaaacaaaggtgaaacaaaccaagaacaagttctcgcaaactgaagcaacctcttatgtacactagatgcttgacttaggaggagtttttaaatgttctcaatgttattctgtagtaaatggcactattatgaagccactagtcattccatatgagtcttaaggactgctctgtgtaacactgtgactgccccgtgtgcttagacacgtagtttcctcagtggatagcactcaacttactccgtagtgatattgtaacaatactgccattccctcttactgcactgcccaacatgtgtgtagcacaaacagttctcattcctaaggaccaattcagaactgaacagtcgcatgtaggacagaaagatacatagaccgggtgtgggagaacacacagcattttgtcaacactgtgcactagtcacatttgtcctgctgccggtagacagccacttcaggaagtgagcctgctacctaacaccgcttctagactcttctcccacttgctattgtggcccgttttcacctccaggtcacagagaatggcaacatcctgaagggagagaccatcttcacatctaccaaaataaaatggaggaatgctaagcatggcctcgtgcttgatctttaggaattagctccgtgttttggacaaaactcaagagaatccccaatagggctggtggtagactttaagcacggggtcggctgctcctcctgcacacacaacacaaaagctaacccctggttgtgattcttccctcatgagagaagaggcaaaccctttgcccttcactcccatcacagcaaactttcagacctagaacagacacacaggacaaggagcaaatccttccctatggatgaacagcacgtttccaacattaaaaccacagatgataggaaacacatactcataggtgagattaaacagcagtttaaacaggagactcaaatgaggggctttcctatctaagggatcaagtcctaccaaagagaaggaacaccttaaataccagacactgacatttaatttcatcatctcccgacttgagttgtacacaatggaacatttccgaggacgcagctccgagctgccgaactgacattacttcctgcattacaatgatactagcacattctcttgcaacactgccaacatgggattgtcaccatagagttagttggtactatatcattctcttgtgagccggtgactggacctgctttctgaccaagatccatcctctgataagccacatgtacctttctgacaatgcagtgtgaagtcttagaagctgatgccctagaaagatcctagttgcctttgtgtatacttactgcctgcttgagtgtttctatgtgtggattttctctgtgtctcgtagaaatgttggggtgttttcttctgccataaggcttgtgacccgcgagccaattcccttagctgtactttcccttcattttttgataagtggtttaaattctgtttcactttgtgtagtgaaccccatggtagttttctgattgttgttaaaaaaaatgacttaacatattacatggacactcaataaaaatgttttatttcctgttga

**Addition files 6: Table S1.** PCR primers for the amplification of the full-length 3’UTR of the *Snap25, Vamp2, Stx1a,* and *Syt1* genes

| Gene name | Primers sequences |
| --- | --- |
| Syt1 | Syt1XhoIF: 5'CCGCTCGAGAGGGAAAACGAAGCCTTTCTGCATCTGCC 3'  Syt1NotIR: 5'ATAAGAATGCGGCCGCTCAACAGGAAATAAAACATTTTTATTGAGTGTC 3' |
|  | mutSyt1F: 5'GACCAATTCAGAACTGAACAGTCGCATGTAGGACAGAAAGATACATAG 3'  mutSyt1R: 5'CTATGTATCTTTCTGTCCTACATGCGACTGTTCAGTTCTGAATTGGTC 3' |
| Stx1a | Stx1aXhoIF: 5'CCGCTCGAGAAACCACCCCACCCACGGCTCCATTCTGG 3'  Stx1aNotIR: 5'ATAAGAATGCGGCCGCTCCAGATACAAATGTTTATTCTACATAAAAATCTC 3' |
|  | mutStx1aF: 5'GAAAAGCCAGGTGTCACTTTGTTAAGTACTTCTCACCCTGCCCTGTCTCC 3'  mutStx1aR: 5'GGAGACAGGGCAGGGTGAGAAGTACTTAACAAAGTGACACCTGGCTTTTC 3' |
| SNAP25 | SNAP25XhoIF: 5'CCGCTCGAG ATCTGCCCTTCTGCTGTGCTCTC 3'  SNAP25NotIR: 5'ATAAGAATGCGGCCGC TTGGGATCTATAATTTTTAATAAT 3' |
|  | mutSNAP25F: 5'CTTTGTTGCTTAATCTAGAGTCGCATGCACCAAATTGCTGAGATGT 3'  mutSNAP25R: 5'ACATCTCAGCAATTTGGTGCATGCGACTCTAGATTAAGCAACAAAG 3' |
| VAMP2 | VAMP2XhoIF:5'CCGCTCGAGGGTTGTCCTTAACACCCCTTCC 3  VAMP2NotIR: 5'ATAAGAATGCGGCCGCTTTACTCATTACACGTTTATTGTACATTT 3' |
|  | mutVAMP2F: 5'CCCCAAGTCCCTTCATTTGCATCTGGATACGTATAGCCCCTCTCCTCCTTCCCTTGG 3'  mutVAMP2R: 5'CCAAGGGAAGGAGGAGAGGGGCTATACGTATCCAGATGCAAATGAAGGGACTTGGGG 3' |

**Addition files 7: Table S2.** Sequences for the Synthesis of *miR-153*, AMO-153 and other various oligonucleotides.

| **oligonucleotides** | Sequences |
| --- | --- |
| *miR-153* mimics | sense: 5’-UUGCAUAGUCACAAAAGUGAUC-3’  antisense:5’-UCACUU UUGUGACUAUGCAAUU-3’ |
| AMO-*miR-153* | 5’-GAUCACUUUUGUGACUAUGCAA-3’ |
| scrambled *miR-153* (mis-*miR-153*) | sense: 5’-UUCUCCGAACGUGCACGUTT-3’ |
|  | antisense: 5’-ACGUGACACGUUCGGAGAATT-3’ |
| scrambled AMO-153 (mis-AMO-153) | 5’-CAGUACUUUUGUGUAGUACAA-3’ |
| *Snap25-ODN* | 5’- G+C+A+A+TTTGGTGTGCATAGC+T+C+T+A-3’ |
| *Vamp2-ODN* | 5’- G+G+G+G+CTATTGCATAGCA+G+A+T+G+C-3’ |
| *Stx1a-ODN* | 5’- G+G+G+T+GAGACTGCAGGCC+C+A+A+A+G-3’, |
| *Syt1-ODN* | 5’- C+T+G+T+CCTATGCATAGCTG+T+T+C+A+G -3 |

*MiR-153* and AMO-153 were synthesized by Shanghai GenePharma Co, Ltd (Shanghai, China). The masking antisense oligodeoxynucleotides (ODNs) of synaptic vesicle-related proteins were synthesized by Shanghai Sangon Biological Engineering Technology and Service Co, Ltd (Shanghai, China). *Snap25-ODN* complements in the position of 1048-1070 containing the binding sites of *miR-153* located in the position 1057-1063 of *Snap25* 3’ UTR; *Vamp2-ODN* masks complements in the position of 313-334 containing the binding sites of *miR-153* located in position *320-327* of *Vamp2* 3’ UTR; *Stx1a-ODN* complements in the position of 836-855 containing the binding sites of *miR-153* located in position 843-848 of *stx-1a* 3’ UTR; *Syt1-ODN* complements in the position of 1615-1635 containing the binding sites of *miR-153* located in position 1622-1628 of *syt1* 3’ UTR. All nucleotides or deoxynucleotides at both ends of the antisense molecules were locked by a methylene bridge connecting between the 2’-O- and the 4’-C atoms.

**Addition files 8: Table S3.** PCR primers for qRT-PCR.

| *miRNAs* | Sequences of primers |
| --- | --- |
| *U6* | RT primer: 5′- CGCTTCACGAATTTGCGTGTCAT-3′  F: 5′- GCTTCGGCAGCACATATACTAAAAT -3′  R: 5′- CGCTTCACGAATTTGCGTGTCAT -3′ |
| *miR-153-3p* | RT primer: 5′-GTCGTATCCAGTGCGTGTCGTGGAGTCGGCAATTGCACTGGATACGACGATCAC-3’  F: 5′- GGGGTTGCATAGTCACAA -3′  R: 5′- CAGTGCGTGTCGTGGAGT -3′ |
| *miR-137-5p* | RT primer: 5‘-CTCAACTGGTGTCGTGGAGTCGGCAATTCAGTTGCTACGCGT -3’  F: 5’-ACACTCCAGCTGGGTTATTGCTTAAGA -3’  R: 5'-CTCAACTGGTGTCGTGGA-3' |
| *miR-34c-5p* | RT primer:5‘-CTCAACTGGTGTCGTGGAGTCGGCAATTCAGTTGGCAATCAG -3’  F: 5‘-ACACTCCAGCTGGGAGGCAGTGTAGT- 3’  R: 5'-CTCAACTGGTGTCGTGGA-3' |
| *miR-135a-5p* | RT primer: 5‘-CTCAACTGGTGTCGTGGAGTCGGCAATTCAGTTGTCACATAG -3’  F: 5‘-ACACTCCAGCTGGGTATGGCTTTTTA- 3’  R: 5'-CTCAACTGGTGTCGTGGA-3' |

**Addition files 9: Table S4.** Antibodies used in the study.

| Antibodies Cat. No. Dilution Source | | | |
| --- | --- | --- | --- |
| Syntaxin-1A | 110 011 | 1:5000 | Synaptic System (Goettingen, Germany) |
| SNAP-25 | 111 011 | 1:5000 | Synaptic System (Goettingen, Germany) |
| Synaptobrein-2 | 104 211 | 1:10000 | Synaptic System (Goettingen, Germany) |
| Synaptotagmin-1 | 105 011 | 1:3300 | Synaptic System (Goettingen, Germany) |
| Munc-13 | 126 111 | 1:1000 | Synaptic System (Goettingen, Germany) |
| Munc-18 | 116 002 | 1:1000 | Synaptic System (Goettingen, Germany) |
| Complexin1/2 | 122 002 | 1:1000 | Synaptic System (Goettingen, Germany) |

**Addition files 8: Table S5.** : Characteristics and cognitive evaluation score of subjects

| Patients | Sex | age | MMSE | MoCA | HAMD | education |
| --- | --- | --- | --- | --- | --- | --- |
| Ctl-1 | male | 75 | 29 | 28 | 2 | bachelor |
| Ctl-2 | male | 73 | 29 | 27 | 3 | bachelor |
| Ctl-3 | male | 74 | 30 | 28 | 3 | bachelor |
| Ctl-4 | male | 87 | 28 | 28 | 2 | bachelor |
| Ctl-5 | male | 86 | 28 | 27 | 0 | bachelor |
| Ctl-6 | male | 89 | 30 | 30 | 2 | bachelor |
| Ctl-7 | male | 70 | 30 | 30 | 0 | master |
| VaD-1 | male | 81 | 28 | 26 | 2 | junior college |
| VaD-2 | male | 81 | 28 | 20 | 4 | junior college |
| VaD-3 | male | 84 | 28 | 25 | 1 | bachelor |
| VaD-4 | male | 83 | 24 | 24 | 3 | bachelor |
| VaD-5 | male | 90 | 29 | 23 | 0 | bachelor |

MMSE: mini-mental state examination; MoCA: Montreal Cognitive Assessment; HAMD: Hamilton Depression Scale

**Additional file 2: Figure S1.**

**
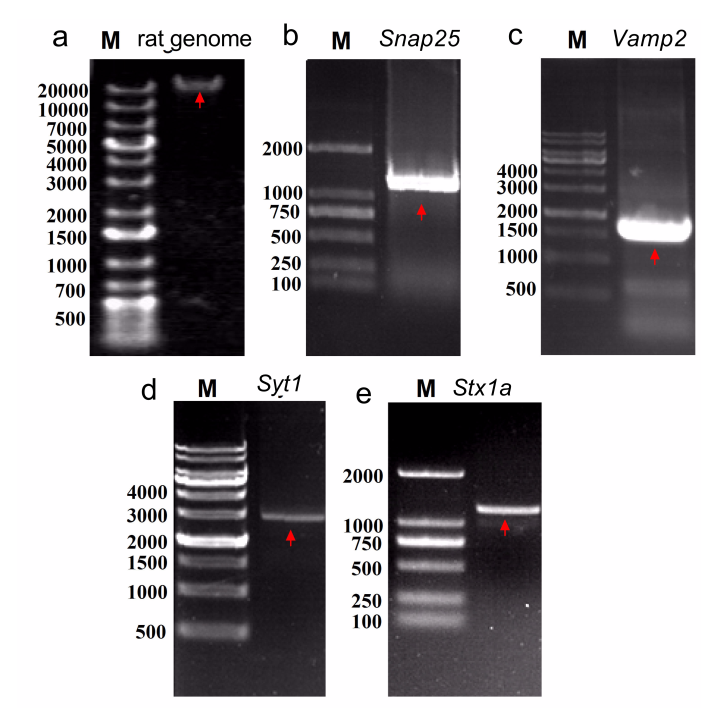
**

**Figure S1.** DNA electrophoresis of extracted rat genome (a), and the full length 3’UTR of *miR-153* targets (red arrow) including *Snap25* (b), *Vamo2* (c), *Syt1* (d) and *Stx1a* (e). M: DNA Ladder Marker.

**Additional file 3: Figure S2.**

**
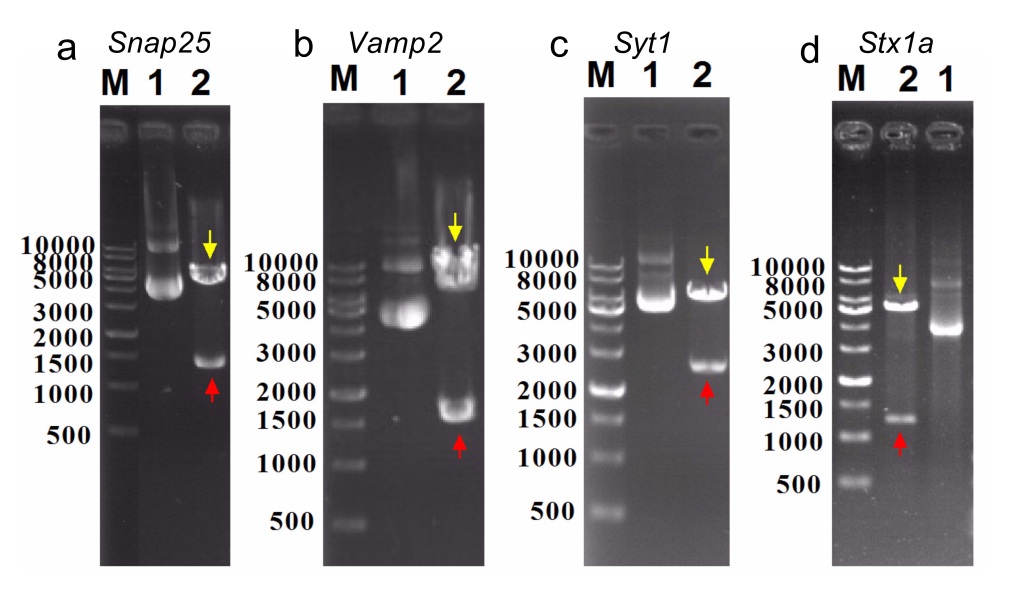
**

**Figure S2.**DNA electrophoresis of plasmid and enzyme digestive vectors of *Snap25* (a)*, Vamp2* (b)*, Syt1* (c)*, Stx1a* (d)*.* M: DL1kb DNA Marker. Yellow arrow: bands of psiCheck vector (6kb); red arrow: bands of target sequences.

**Addition files 4: Figure S3:**

**
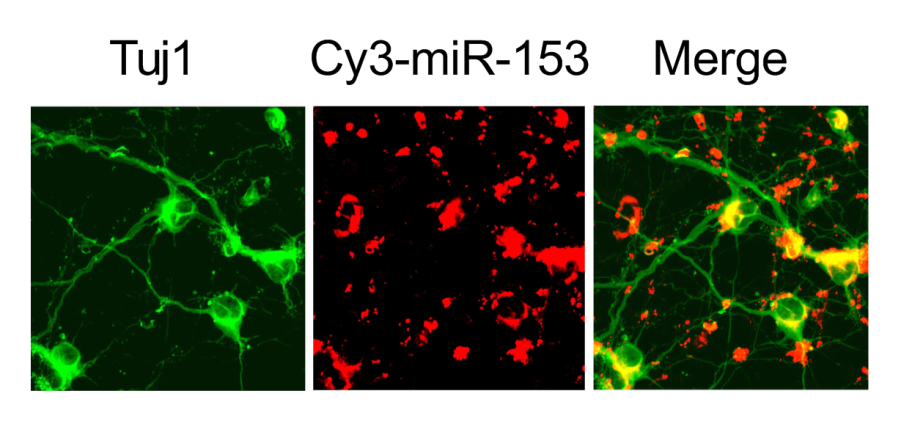
**

**Figure S3:** Identification of transfection of Cy3-labelled *miR-153* mimics (Cy3-*miR-153*) by X-treme into the NRNs. Green: Tuj1; Red: Cy3-*miR-153*.

**Addition files** 5**: Figure S4**

**
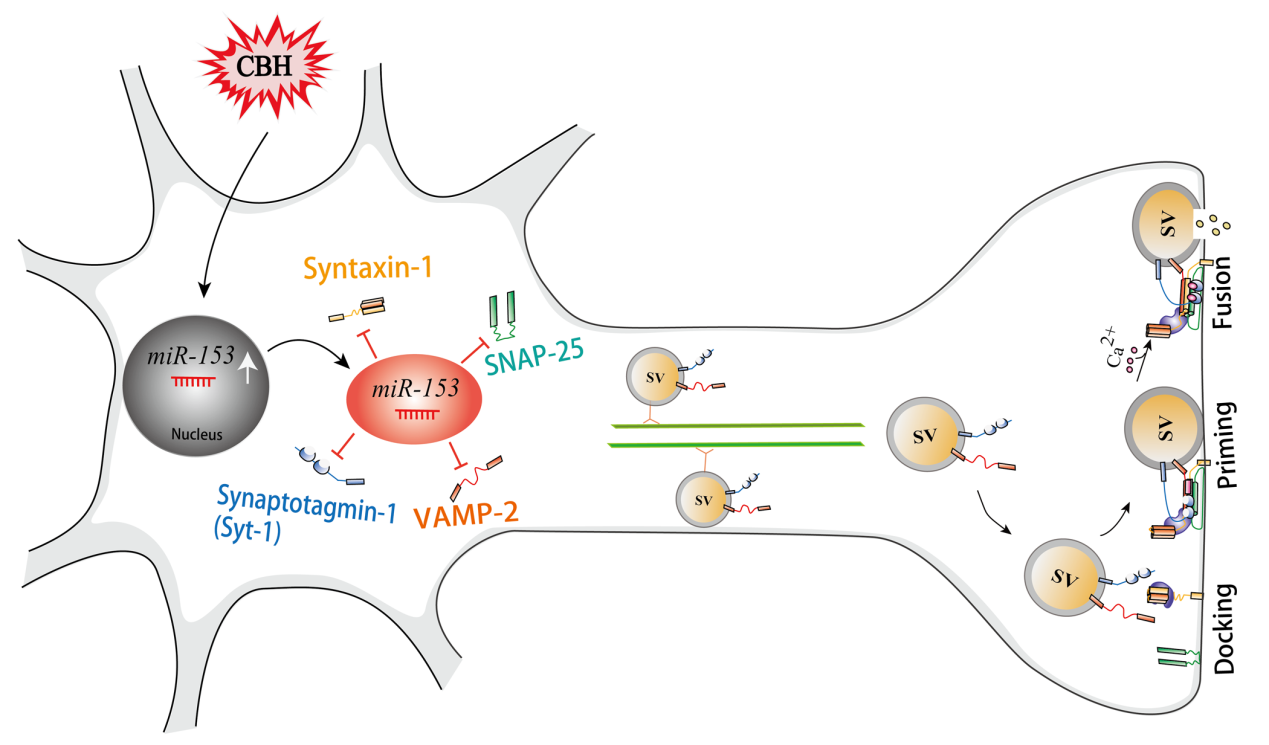
**

**Figure S4** Schematic diagram of the *miR-153-*mediated impairment in presynaptic vesicle release in the hippocampus of rat following CBH.

CBH resulted in brain hypoxia, which induced *miR-153* upregulation, which post-transcriptionally regulates the expression of vesicle-related proteins by binding the 3’UTRs of the *Stx1a*, *Snap25*, *Vamp2* and *Syt1* genes, leading to impaired presynaptic vesicle release by disturbing synaptic vesicle fusion with the presynaptic membrane. SNAREs: soluble *N* - ethylmaleimide - sensitive factor attachment; VAMP-2: vesicle associated membrane protein 2; SNAP-25: synaptosomal-associated protein 25.
